# Sirtuin4 alleviates severe acute pancreatitis by regulating HIF-1α/HO-1 mediated ferroptosis

**DOI:** 10.1038/s41419-023-06216-x

**Published:** 2023-10-21

**Authors:** Yanna Liu, Huning Cui, Chaopeng Mei, Mengwei Cui, Qianqian He, Qiaofang Wang, Dejian Li, Yaodong Song, Jiye Li, Sanyang Chen, Changju Zhu

**Affiliations:** 1 Department of Emergency, The First Affiliated Hospital of Zhengzhou University, No 1 Eastern Jianshe Road, Zhengzhou, 450052 Henan China; 2 Henan Medical Key Laboratory of Emergency and Trauma Research, Zhengzhou, Henan 450052 China; 3 Henan Emergency and Trauma Medicine Engineering Research Center, Zhengzhou, Henan 450052 China; 4 Translational Medical Center, The First Affiliated Hospital of Zhengzhou University, Zhengzhou, Henan 450052 China

**Keywords:** Cell signalling, Cytokines

## Abstract

Acute pancreatitis (AP) is a common emergency of the digestive system and serious cases can develop into severe acute pancreatitis (SAP), which ortality rates up to 30%. Sirtuin4 (SIRT4) is a member of the sirtuin family, and plays a key role in inflammation and oxidative stress. However, the potential role of SIRT4 in SAP has yet to be elucidated. In the present study, we found that the expression level of SIRT4 in human AP was downregulated by screening a public database, suggesting that SIRT4 may play a role in AP. Subsequently, we used L-arginine (L-Arg) to induce SAP in SIRT4 knockout (SIRT4_KO) and SIRT4 overexpression (AAV_SIRT4) mice. The results showed that the pancreatic tissue injury and related lung and kidney injury were serious in SIRT4_KO mice after SAP induction, but were significantly reduced in AAV_SIRT4 mice. More importantly, we found that the levels of antioxidant factors GSH and SOD were decreased in SIRT4_KO mice, and the production of oxidative products and lipid peroxidation markers was increased, suggesting that SIRT4 was involved in inflammation and oxidative stress during SAP. Further studies showed that the absence or overexpression of SIRT4 affected the expression level of Hypoxia-inducible factor-1α (HIF-1α) after SAP induction, and regulated the expression of ferroptosis related proteins by mediating HIF-1α/HO-1 pathway. Collectively, our study revealed that SIRT4 plays a protective role in SAP by regulating the HIF-1α/HO-1 pathway to inhibit ferroptosis.

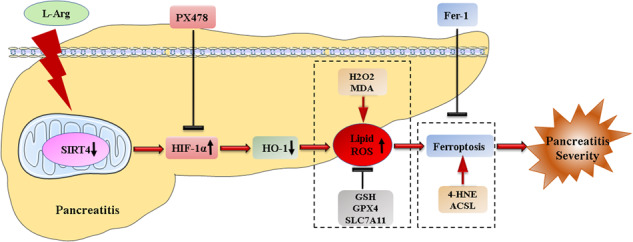

## Introduction

Acute pancreatitis (AP) is a form of pancreatic autodigestion caused by enzymic activation. AP develops into systemic inflammation while local inflammation in the pancreas can develop into persistent systemic inflammatory response syndrome (SIRS) which can subsequently lead to multiple organ dysfunction syndrome (MODS) [1, 2]. Approximately 20% of AP patients have severe and complex pancreatitis, which subsequently develops into severe acute pancreatitis (SAP); this condition has a mortality rate exceeding 30% [3]. The therapeutic measures for SAP are limited, so it is necessary to further study the molecular mechanism and explore effective therapeutic targets.

AP is caused by local aseptic inflammation and damage to the pancreatic tissue caused by the premature activation of pancreatic enzymes [4]. Inflammatory reactions play an important role in the outcome of AP. Macrophages and neutrophils infiltrate and release cytokines such as interleukin-6 (IL-6) and tumor necrosis factor-α (TNF-α) to promote the inflammatory response and cause serious complications such as SIRS [5, 6]. There is a dynamic balance between oxidation and antioxidation in normal pancreatic tissue [7]. The production of reactive oxygen species (ROS) increases with the decreasing capacity for systemic clearance during SAP, thus resulting in the accumulation of ROS in pancreatic tissue. This can cause direct damage to cells or cause a series of pathophysiological changes by inducing the production of inflammatory mediators [8].

Ferroptosis is a form of iron-catalyzed and oxidative cellular necrosis, that is mainly manifested by iron accumulation and the extensive production of lipid peroxide and ROS. Activation of ferroptosis requires acyl-CoA synthetase long-chain family member 4 (ACSL4) and lysophosphatidylcholine acyltransferase 3 (LPCAT3). The antioxidant system, including glutathione (GSH), solute carrier family 7 member 11 (SLC7A11) and glutathione peroxidase 4 (GPX4), could interrupt the occurrence of lipid peroxidation and inhibit the progress of ferroptosis [9]. It is possible that an inhibitor of ferroptosis, such as ferrostatin-1 (Fer-1), could not only restrain the production of ROS, but also inhibit the release of cytokines to reduce the inflammatory response, thus exerting an anti-inflammatory role [10]. Previous studies suggest that ferroptosis may also be the factor that initiates inflammation [11]. Therefore, there is a close relationship between ferroptosis and inflammation. SAP is known to be associated with abnormal iron metabolism and increased ROS production; therefore, the inhibition of ferroptosis has the potential to alleviate pancreatitis and associated organ damage [12, 13].

The sirtuin family is an NAD+ dependent protease family composed of SIRT1-7 members, which play an important role in pyrodeath and oxidative stress[14–16]. Previous studies have reported that SIRT4 is associated with numerous diseases that involve mitochondrial dysfunction, including non-alcoholic diseases, type 2 diabetes, fatty liver, apoptosis, inflammation, vascular diseases and the development of various cancers [17]. SIRT4 is an important regulator of inflammation and controls the expression of cytokines and the levels of oxidative stress [18–21]. However, the role of SIRT4 in SAP remains unknown. In this study, we aimed to investigate the role of SIRT4 in SAP and to evaluate whether SIRT4 could protect against SAP and associated lung/kidney injury and prevent ferroptosis.

In our study, we first used a public database to determine the expression levels of SIRT4 in human AP. Then, we used L-arginine (L-Arg) to construct a mouse model of SAP and an in vitro model of SAP in pancreatic acinar AR42J cells. In addition, we used SIRT4 knockout (SIRT4_KO) mice and adeno-associated virus-mediated SIRT4 overexpression (AAV_SIRT4) mice, and constructed a SIRT4 knock-down (SIRT4 KD) plasmid and SIRT4 overexpression plasmid (SIRT4 OE) to transfect AR42J cells for experiments. Both in vivo and in vitro experiments demonstrated that SIRT4 plays an important protective role in SAP. SIRT4 was shown to alleviate inflammatory reactions, oxidative stress and ferroptosis in SAP. Further analysis showed that SIRT4 regulate inflammation and oxidative stress to inhibit ferroptosis and alleviate SAP via the HIF-1α/HO-1 pathway.

## Materials and methods

### Reagents

L-Arg and BCA protein assay kits were purchased from Solebao Biotechnology Co., Ltd. (Beijing, China). Adenoad-associated viruses (AAV) and plasmid were both purchased from Hanheng Biotechnology Co., Ltd. (Shanghai, China). SIRT4 siRNA was purchased from genepharma (Shanghai, China). The FOREGENE mouse tail gene identification box was purchased from Chengdu Fuji Biotechnology Co., Ltd. (Chengdu, China). The ferroptosis inhibitor Fer-1 (HY-100579) and HIF-1α inhibitor PX478 (HY-10231) were both purchased from MedChemExpress Corporation (Shanghai, China). The amylase ELISA kit (mlo27545) was purchased from Shanghai Enzyline Biotechnology Co., Ltd. (Shanghai, China). The lipase ELISA kit (A054-2-1) was purchased from the Nanjing Construction Institute of Biological Engineering (Nanjing, China). The MCP-1 ELISA kit (EMC113.96), IL-6 ELISA kit (EMC004.96), IL-1β ELISA kit (EMC001b.96) and TNF-α ELISA kit (EMC102a.96) of mouse were all purchased from NeoBioscience Technology Co., Ltd. (Shenzhen, China). MDA kit (A003-1-2), Total SOD kit (A001-1-1), trace and prototype GSH kit (A006-2-1) were purchased from Nanjing Construction Institute of Biological Engineering (Nanjing, China). ROS determination kit (S0033) and hydrogen peroxide (H_2_O_2_) detection kit (S0038) was purchased from Beyotime Biotechnology Co., Ltd. (Shanghai, China). Dulbecco’s-modified Eagle’s medium (DMEM) and fetal bovine serum (FBS) were purchased from Gibco (Thermery Fisher Scientific, Waltham, MA, USA). Primary antibodies were purchased from the following companies: glyceraldehyde-3-phosphate dehydrogenase (GAPDH) (Beyotime, Shanghai, China), heme oxygenase-1 (HO-1), solute carrier family 7 member 11 (SLC7A11) and Hypoxia-inducible factor-1α (HIF-1α) (Proteintech, Wuhan, China), NADPH-quinone oxidoreductase1 (NQO-1) (HuaBio, Hangzhou, China), glutathione peroxidase4 (GPX4), acyl-CoA synthetase long-chain family member4 (ACSL4) and Sirtuin4 (SIRT4) (abclonal, Wuhan, China), lymphocyte antigen 6 complex locus G6D (Ly6G) and myeloperoxidase (MPO) (Abcam, Shanghai, China), cluster of differentiation molecule 11b (CD11b) (Boster, Wuhan, China), 4-hydroxynoneral (4-HNE)(RD systems, Shanghai, China).

### Dataset analysis from the GEO database

The NCBI gene expression omnibus (GEO) database is an open genomics database that collects submitted high-throughput gene expression data. A thorough query was conducted on all data sets involving AP research. Studies are considered eligible for subsequent analysis based on the following criteria: studies of pancreatic tissue or blood samples from patients with AP; provide information about the technologies and platforms used for research; a control study using the normal group. According to these standards, a dataset GSE194331 (containing 87 AP samples and 32 normal samples) [22] on the AP was downloaded from the repository. GraphPad Prism was used to analyze gene expression differences between the SIRT4 gene expression data of two groups of samples.

### Animal

Male C57BL/6 N mice about 6-8 weeks old weighing 18–22 g were all from the Huaxing Experimental Animal Center (Zhengzhou, China). Mice were maintained at laboratory condition under a 12-hour light/dark cycle at 25 ± 2 °C with 50 ± 5% relative humidity and given free access to food and water. Male and female SIRT4 heterozygous (SIRT4 + /−) mice (C57BL/6 background) were obtained from the Cyagen Biotechnology Co., Ltd. (Jiangsu, China). And mice were acclimatized for one week prior to mating and breeding procedures. The genomic DNA of the mouse tail tip was extracted for genotyping. The identified SIRT4_KO mice were fed for 4 weeks for before experiments. C57BL/6 mice were injected intraperitoneally with a single dose of AAV9-SIRT4 to overexpress SIRT4 or control vectors (2 × 10^11^ viral genomes (VG) /mouse, diluted to 200 μl in sterile PBS) to obtain adeno-associated virus-mediated SIRT4 overexpression (AAV_SIRT4) mice or empty adeno-associated virus (AAV_GFP) mice. Animal models were constructed after three weeks of normal feeding. All the studies were approved by the Ethical Committee of the First Affiliated Hospital of Zhengzhou University (Ethical Review Number 2022-KY-0051-001).

### Animal model

All mice fasted for 12 h and drank freely before the experiment. The L-Arg powder is first dissolved in normal saline, and then adjusted to pH 7 with 10% hydrochloric acid. The SAP model was established by intraperitoneal injection of L-Arg (4 g/kg) twice with an interval of an hour [23]. The animals were treated with an equivalent amount of normal saline to replace L-Arg in sham group. After injection, the mice ate and drank freely. The mice were anesthetized with 1% pentobarbital and sacrificed 72 h after the last injection. Mice eyeball blood sample were collected and centrifugated at 4 °C for 10 min at 3000 g, and the serum was separated and then stored at -80 °C for future investigation. Pancreatic tissue, lung tissue and kidney tissue were harvested and fixed in 4% Polyformaldehyde solution for histological analysis, and the rest of them were stored at -80 °C for future use. Fer-1 (10 mg/kg) and PX478 (100 mg/kg) were intraperitoneally injected in mice an hour prior L-Arg injection [24, 25].

### Cell culture and treatment

Rat pancreatic acinar AR42J cells were cultured in F12K medium containing fetal bovine serum in a 37 °C incubator containing 5% CO_2_. Conduct relevant experiments when the cell growth rate reaches about 90%. AR42J cells were treated with L-Arg (10 mg/ml) for 24 h to establish SAP model in vitro, while the CON group only treated with PBS solution as a control, and then cell samples were collected for subsequent experiments.

SIRT4 siRNA and SIRT4 overexpression plasmid were constructed to transfect AR42J cells with lipofectamine 3000 transfection reagent to knock down or overexpress SIRT4 according to the instructions. Cells were incubated with L-Arg (10 mg/ml) to establish SAP model in vitro after 24 h of transfection.

### Determination of serum amylase, lipase levels and trypsase activity

Serum amylase and lipase level, trypsase activity were evaluated by enzyme-linked immunosorbent assay (ELISA) using commercially available kits in accordance with manufacturer’s instructions.

### Oxidative stress and inflammatory factor assays

The content of GSH, malondialdehyde (MDA), superoxide dismutase (SOD), hydrogen peroxide (H_2_O_2_) in pancreatic tissue were detected with the corresponding kit according to the instructions. The levels of tumor necrosis factor-α (TNF-α), Monocyte chemoattractant protein-1 (MCP-1), interleukin-1β (IL-1β) and interleukin-6 (IL-6) in serum were determined by ELISA kit.

### Hematoxylin and eosin (HE) staining and immunohistochemistry (IHC)

The pancreas, lung and kidney tissues were embedded in paraffin and cut into 4 μM serial sections and then stained with HE. The pathological changes such as edema, inflammation and necrosis were observed with a microscope, cells with swelling of the cytoplasm, loss of plasma membrane integrity, and leakage of organelles into the stroma are considered necrotic. Pathological grading of pancreatic tissue was performed according to Schmidt’s scoring criteria [26, 27], with a score of 0–4. A score of 0–3 was obtained based on the degree of edema, leukocyte infiltration, and bleeding in the lung tissue [28]. According to the percentage of renal tubular necrosis, tubular dilation, and brush border loss in the picture, the degree of renal injury was rated as 0–5 [29]. Pancreatic tissue sections were stained with IHC for cluster of CD11b, MPO, LY6G, 4-HNE, and then observed with microscope.

### Determination of ROS

According to the instructions of ROS test kit, AR42J cells were stained with 2,7-Dichlorodihydrofluorescein diacetate (DCFH-DA). Fluorescence microscope (Olympus DX51) was used to observe.

### Cell viability assay

AR42J cells were seeded in 96-well plates at 8 × 10^3^ cells/well density. The SAP model was initiated when the cell density reached 80% confluence. After the treatment, 10 μL of CCK-8 reagent (Boster, Wuhan, China) was added to each well according to the CCK-8 kit guidelines. The absorbance of each well was measured at 450 nm using a microplate reader after the plates were incubated at 37 °C for 1 h. The cellular activity was then assessed based on the absorbance values obtained.

### Protein extraction and western blot

RIPA lysis buffer, protease inhibitor and phosphatase inhibitor were used to separate proteins from pancreatic tissues. BCA kit was used to detect total protein concentration. The protein samples were added into the prepared gel pore to conduct electrophoretic separation, and then transferred to the polyvinylidene fluoride (PVDF) membrane. Then, the membrane was put into the quick sealing solution and sealed at room temperature for 15 min, then washed with TBST solution for three times (5 min each time). Then the membrane was incubated with GAPDH (1:3000), ACSL4 (1:1000), SLC7A11 (1:1000), HO-1 (1:1000), NQO-1 (1:1000), SIRT4 (1:1000), HIF-1α (1:1000) and GPX4 (1:1000) overnight at 4 °C on a shaking table. After washing with TBST solution for three times (5 min each time), the membrane was incubated with the anti-rabbit or mouse IgG secondary antibody (1:5000) coupled with HRP in a shaking table at room temperature for 1 h. Again, the membrane was washed three times (5 min/time) with TBST solution. Chemiluminescent solution was dropped on the membrane, and the image was acquired using the Odyssey imaging system (LI-COR, Linkon, NE, USA). Protein abundance was assessed in at least three biological replicates. The bands were quantified by ImageJ software (NIH, Bethesda, MD, USA), and GAPDH was used as control.

### Real-time (RT)-PCR

Total RNA was isolated using TRIzol reagent (Servicebio, Wuhan, China). Two micrograms of total RNA were reverse transcribed into complementary DNA (cDNA) using Reverse Transcription kit (Vazyme, Nanjing, China). The GAPDH gene was used as an internal housekeeping control. Primers used are listed in Supplementary Table 2.

### Extraction of mitochondrial proteins

Using the Cell Mitochondrial Isolation kit (C3601), we extract mitochondrial proteins by collecting cells, washing cells, pretreatment, homogenizing, centrifugation, and removing the supernatant according to the instructions.

### Statistical analysis

GraphPad Prism (version 8.0.2) was used for all statistical analyses. All data analyzed in this study were presented as the mean ± standard deviation (SD). The two groups were compared by independent sample t-test and three or more groups were compared by one-way analysis of variance (ANOVA) plus Tukey’s multiple comparison test or Kruskal-Wallis nonparametric test. The *p* values less than 0.05 was considered statistically significant.

## Results

### SIRT4 expression was downregulated in SAP

**Fig. 1:**
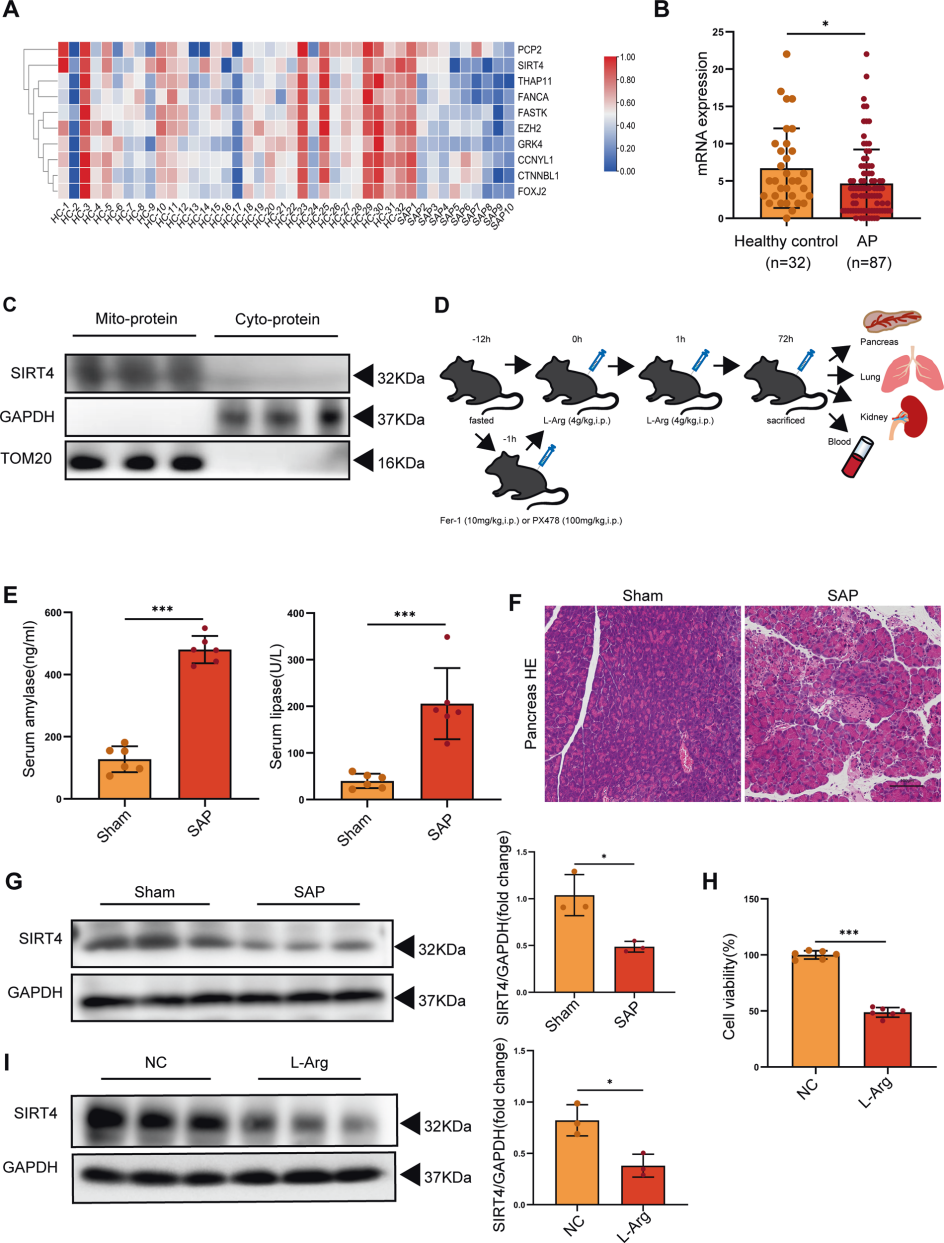
SIRT4 expression was down-regulated in SAP. **A** The correlation between SIRT4 and AP was discovered by bioinformatics analysis. **B** SIRT4 mRNA expression was downregulated in AP patients (GEO dataset GSE194331). **C** SIRT4 is localized in mitochondria. **D** Animal SAP model and experimental protocol for drug (Fer-1, PX478) administration. **E** Serum amylase level and lipase activity. **F** Typical HE staining images of pancreatic tissue. Scale bar = 100 µm. **G** Protein expression level of SIRT4 in the pancreas. **H** Viability of AR42J cells. **I** The protein expression level of SIRT4 in AR42J cells. Sham group (saline), SAP group (4 g/kg L-Arg), NC group (PBS), L-Arg group (10 mg/ml L-Arg). **E**, **G** used GAPDH as the reference protein. *P < 0.05, ***P < 0.001. *n* = 6 per group.

By analyzing an original microarray data set, we found that SIRT4 was down-regulated in AP patients when compared with healthy subjects (Fig. 1A, B). In addition, we verified SIRT4 protein expression in mitochondria by extracting mitochondrial protein for Western Blot (Fig. 1C, Supplementary Fig. 1C). In order to analyze the relationship between SIRT4 and SAP, we used L-Arg to induce SAP in WT mice (Fig. 1D). The results showed that the serum amylase level and lipase activity of mice in SAP group were higher than those in sham group (Fig. 1E), and HE staining also showed that the degree of pancreatic tissue edema, inflammatory cell infiltration and necrosis of mice in SAP group was significantly greater than that in sham group (Fig. 1F). However, SIRT4 expression in pancreatic tissues was significantly down-regulated (Fig. 1G, Supplementary Fig. 1G). In vitro experiments were consistent with the above results. Compared with the NC group, L-Arg treatment significantly reduced the viability of AR42J cells and the expression level of SIRT4 (Fig. 1H, I, Supplementary Fig. 1I). These results suggest that SIRT4 expression is down-regulated in SAP and affects the severity of SAP.

### SIRT4 plays a key role in SAP mice and influences SAP-related lung and kidney injury

**Fig. 2:**
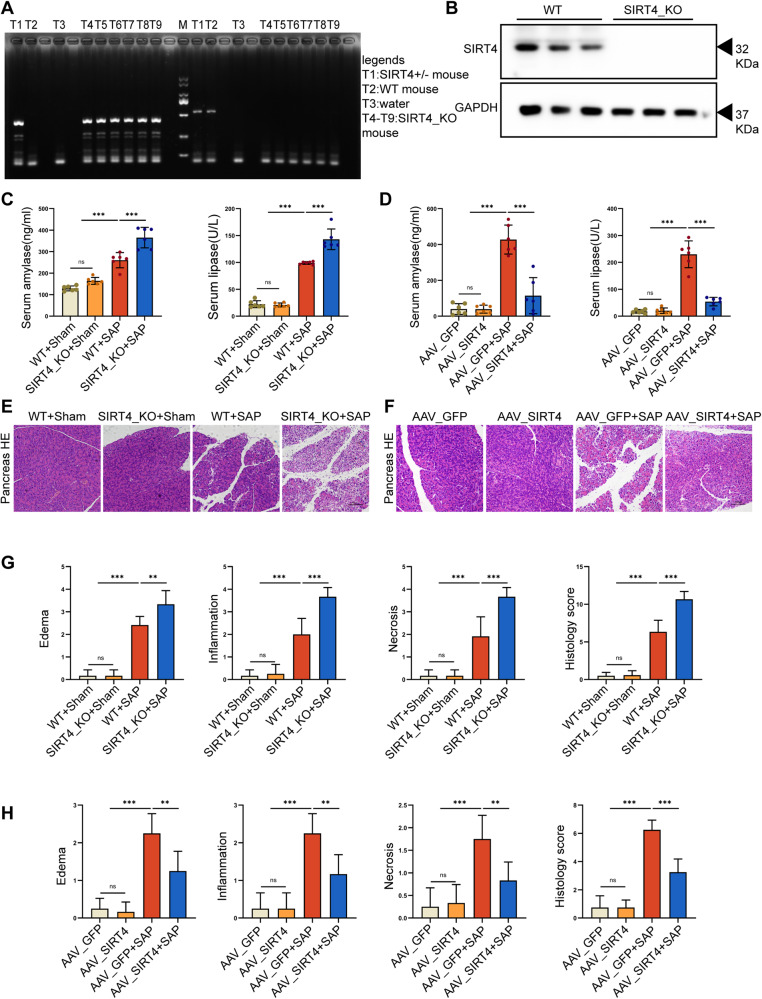
The pivotal role of SIRT4 in SAP mice. **A** Mouse tail gene identification kit was used for gene identification, M was DNA Maker, T1 was SIRT4 heterozygous (SIRT4 +/-) mice, T2 was WT mice, T3 was water, T4-T9 was SIRT4_KO mice. **B** Protein levels of SIRT4 in the pancreas. **C**–**D** Serum amylase level and lipase activity. **E**–**F** Typical HE staining images of pancreatic tissue. Scale bar = 100 µm. **G**–**H** Statistics of pancreatic pathological damage. WT group (wild type mice), SIRT4_ KO group (SIRT4 knockout mice), WT+Sham group (WT+saline), SIRT4_KO+ Sham group (SIRT4_KO+saline), WT + SAP group (WT + 4 g/kg L-Arg), SIRT4_KO + SAP group (SIRT4_KO + 4 g/kg L-Arg), AAV_GFP group (empty virus+saline), AAV_SIRT4 group (SIRT4 overexpression+saline), AAV_GFP + SAP group (AAV_GFP + 4 g/kg L-Arg), AAV_SIRT4 + SAP group (AAV_SIRT4 + 4 g/kg L-Arg). B used GAPDH as the reference protein. **P < 0.01, ***P < 0.001, ns. not significant. *n* = 6 per group.

SIRT4 knockout (SIRT4_KO) mice were used to further investigate the role of SIRT4 in SAP. SIRT4_KO mice were obtained by targeted reproductive activity and genotyping was performed using genomic DNA isolated from the mouse tail tip to confirm gene deletion (Fig. 2A, B, Supplementary Fig. 2B). Compared with the control group, serum amylase and lipase activities were increased in SIRT4_KO mice after SAP induction; however, serum amylase and lipase activities were significantly decreased in SAP mice injected with overexpression of adeno-associated virus SIRT4 (AAV_SIRT4) (Fig. 2C, D). Pancreatic tissue edema, inflammatory cell infiltration, necrosis, and pathological scores also showed consistent results, namely, the SIRT4_KO group was greater than the control group, while the AAV_SIRT4 group was significantly less than the control group (Fig. 2E–H). These results suggest that the loss of SIRT4 exacerbates SAP, while SIRT4 overexpression improves pancreatic injury.

**Fig. 3:**
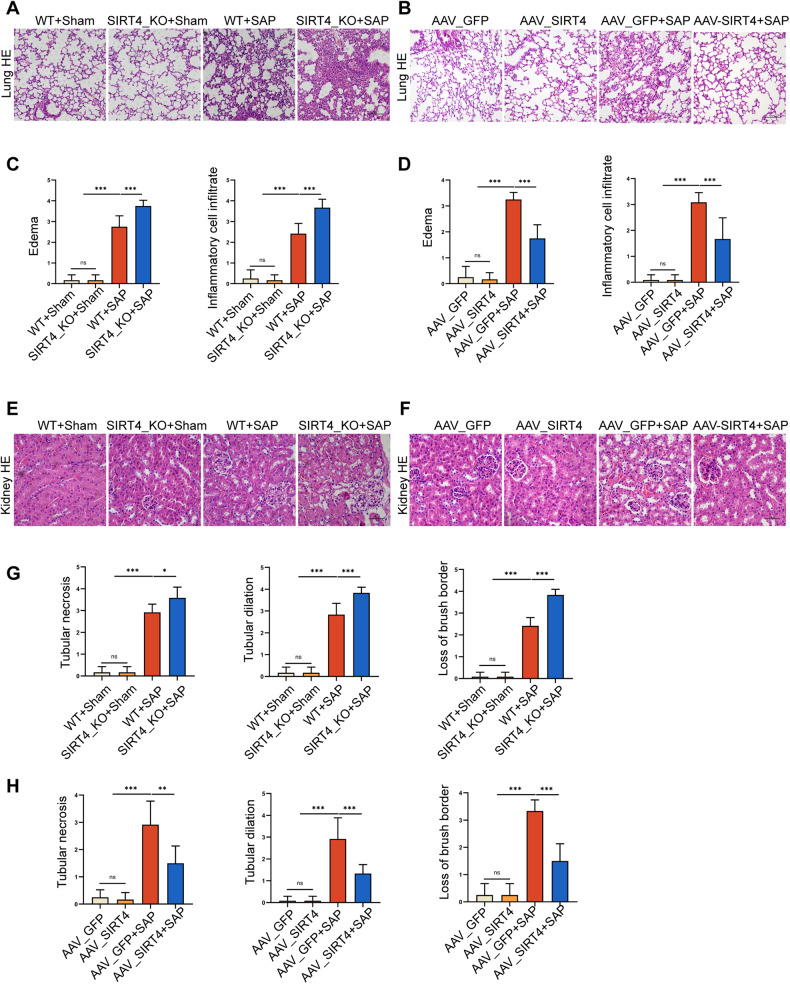
SIRT4 deficiency aggravated SAP-related lung and kidney injury in mice. **A**–**B** Representative histological HE staining images in lung of mice. Scale bar = 100 µm. **C**–**D** Statistics showing pathological injury in the lung of mice. **E**–**F** Representative histological HE staining images in kidney of mice. Scale bar = 50 µm. **G**–**H** Statistics showing pathological injury in the kidney of mice. *P < 0.05, **P < 0.01, ***P < 0.001, ns. not significant*. n* = 6 per group.

During the development of SAP, a range of related organs may be damaged. We then examined common SAP-related lung injury and kidney injury. HE staining was performed on the lung and kidney tissues of mice induced by SAP, and the results showed that the lung and kidney tissues of mice were damaged. In addition, compared with the control group, the SAP group showed obvious edema and inflammatory cell infiltration in the lungs, while the SIRT4_KO group increased edema and inflammatory cell infiltration, while the AAV_SIRT4 group significantly reduced lung injury (Fig. 3A–D). Renal injury also showed consistent results. Compared with the control group, tubule dilatation and necrosis, brush boundary loss, and remodeling were heavier in the SAP group, and heavier in the SIRT4_KO group; however, kidney injury was significantly reduced in the AAV_SIRT4 group (Fig. 3E–H). Furthermore, we found that SIRT4 overexpression could increase the expression level of SIRT4 in the pancreas after protein level and mRNA level, and had almost no effect on the expression level of SIRT4 in the lung and kidney, suggesting that SIRT4 overexpression did not affect lung and kidney pathology (Fig. S1A, B, Supplementary Fig. S1A). These results suggest that SIRT4 deficiency aggravates SAP-related lung injury and kidney injury in mice.

### SIRT4 regulated inflammatory response and oxidative stress during SAP

**Fig. 4:**
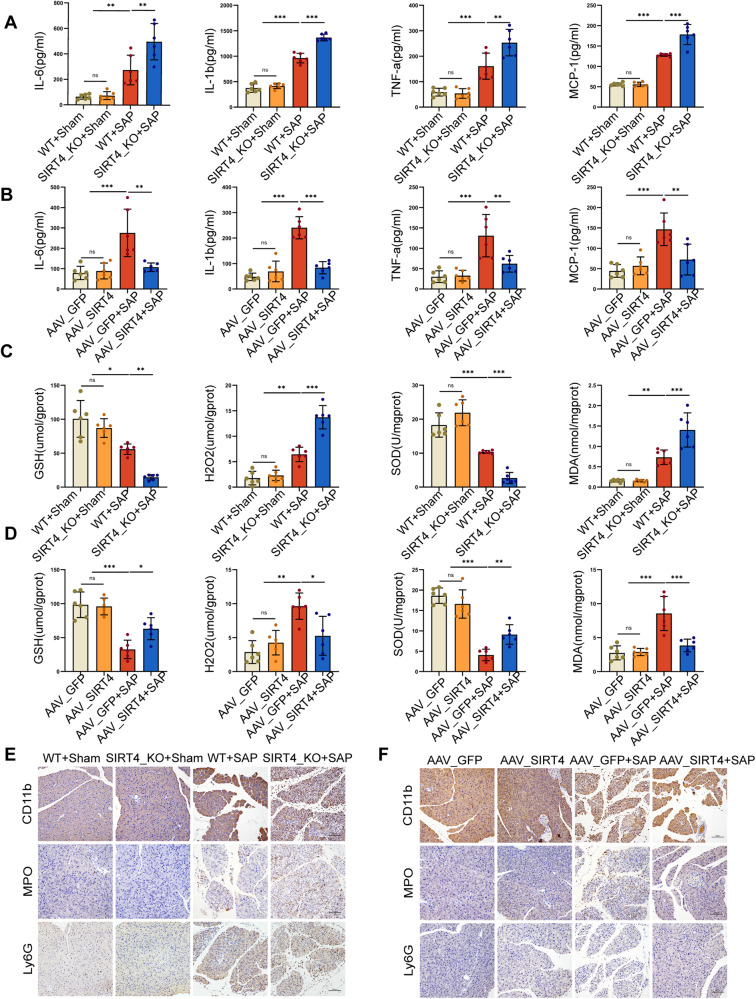
SIRT4 regulated inflammatory response and oxidative stress during SAP in mice. **A**–**B** Statistics for the levels of inflammatory factors (IL-6, IL-1β, TNF-α and MCP-1). **C**–**D** Statistics for the levels of oxidative stress (GSH, H_2_O_2_, SOD and MDA) in the pancreas of mice. **E**–**F** Representative immunohistochemical staining of CD11b, MPO and Ly6G in the pancreas of mice. Scale bar = 100 µm. *P < 0.05, **P < 0.01, ***P < 0.001, ns. not significant. *n* = 6 per group.

Next, we measured the levels of serum inflammatory factors and oxidative stress indicators in pancreatic tissue. Compared to the SAP group, the levels of inflammatory factors (IL-6, IL-1β, TNF-α and MCP-1) in the serum of SIRT4_KO mice were increased (Fig. 4A), the levels of antioxidants (GSH and SOD) were decreased, and the oxidation product H_2_O_2_ along with the production of the lipid peroxidation marker MDA were increased (Fig. 4C). Ly6G is a specific marker of neutrophils, MPO is a marker of functional activation of neutrophils, and CD11b marks immune cells such as monocytes, macrophages, neutrophils, and NK cells. IHC staining and quantitative analysis showed that the expression levels of Ly6G, CD11b and MPO in pancreatic tissue were significantly increased, thus indicating increased inflammatory cell infiltration (Fig. 4E, Fig. S2C). Similarly, in AAV_SIRT4 mice with SAP, the levels of inflammation (Fig. 4B) and oxidative stress (Fig. 4D) were reduced; IHC staining and quantitative analysis indicated that inflammatory cell infiltration was also reduced (Fig. 4F, Fig. S2D). These findings indicated that SIRT4 regulated inflammation and the levels of oxidative stress level in SAP.

**Fig. 5:**
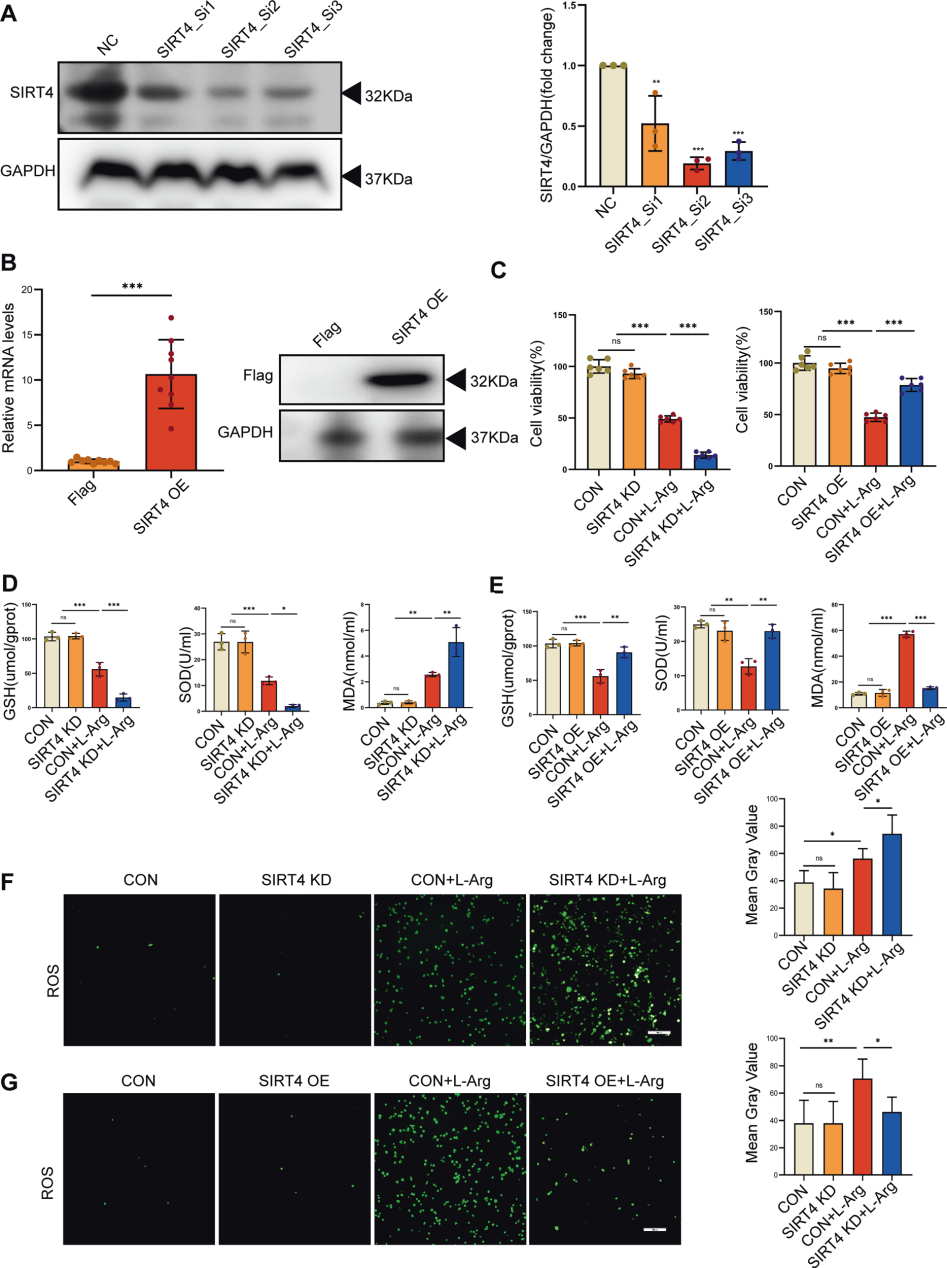
SIRT4 mediated oxidative stress in SAP in AR42J cells. **A** Western blot showing the SIRT4 knock down efficiency. **B** Transfection efficiency of SIRT4 overexpressed using the Flag plasmid in AR42J cells. **C** Viability of AR42J cells. **D**–**E** The levels of oxidative stress (GSH, SOD and MDA) in AR42J cells. **F**–**G** Representative images showing ROS in the AR42J cells. Scale bar = 100 µm. CON group (PBS), SIRT4 KD group (SIRT4 knockdown), CON + L-Arg group (10 mg/ml L-Arg), SIRT4 KD + L-Arg group (SIRT4 KD + 10 mg/ml L-Arg), SIRT4 OE group (SIRT4 overexpression), SIRT4 OE + L-Arg group (SIRT4 OE + 10 mg/ml L-Arg). A used GAPDH as the reference protein. *P < 0.05, **P < 0.01, ***P < 0.001, ns. not significant. *n* = 6 per group.

In order to further clarify the protective effect of SIRT4 on SAP, we used L-Arg and a rat pancreatic acinar AR42J cell line to establish a model of SAP in vitro. We used three types of siRNA to knock down SIRT4. According to the results of Western blot (Fig. 5A, Supplementary Fig. 5A), we selected the siRNA with the highest knockdown efficiency for subsequent experiments. We confirmed successful transfection by two methods. First, we verified the overexpression of SIRT4 mRNA by PCR experiments, and confirmed that SIRT4 overexpression transfection was successful. Secondly, we verified the transfection efficiency of SIRT4 overexpression of Flag plasmid by WB experimental method (Fig. 5B, Supplementary Fig. 5B). We found that the survival of pancreatic acinar cells featuring SIRT4 knockdown was significantly reduced after L-Arg stimulation (Fig. 5C), the levels GSH and SOD were decreased, the levels of the lipid peroxidation product MDA were increased (Fig. 5D), and the levels of cellular ROS were increased (Fig. 5F). As we expected, the overexpression of SIRT4 had a protective effect on AR42J cells when stimulated by L-Arg, with increased cell survival (Fig. 5C), increased antioxidant levels, reduced MDA levels (Fig. 5E), and reduced ROS production (Fig. 5G). In addition, we also studied the difference in ROS production and expression of digestive enzymes, such as amylase protein expression levels and cell activity, in the experimental models of CER and CER + LPS (Fig. S1C, D, Supplementary Fig. S1D). Therefore, our in vitro experiments demonstrated that SIRT4 regulates oxidative stress in SAP.

### SIRT4 regulated ferroptosis in vivo and in vitro

**Fig. 6:**
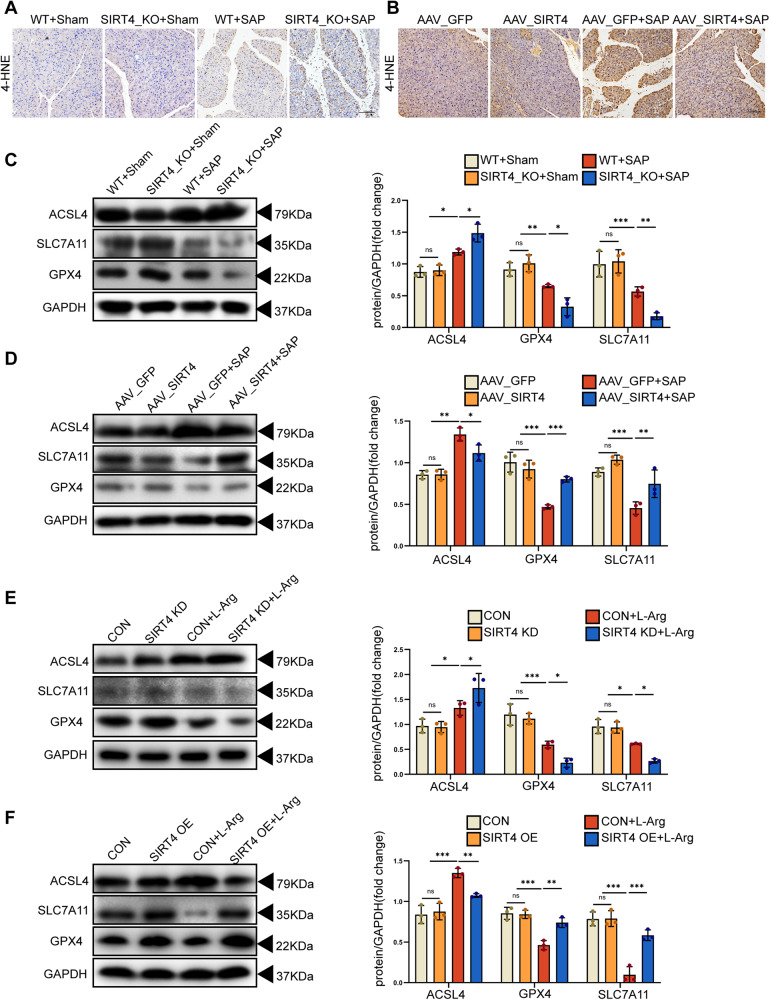
SIRT4 regulated ferroptosis in vivo and in vitro. **A**–**B** Representative IHC staining of 4-HNE in the pancreas of mice. Scale bar = 100 µm. **C**–**D** Western blot of different types of ferroptosis-related proteins (ACSL4, GPX4, SLC7A11) in pancreas of mice. **E**–**F** The protein expression level of ferroptosis-related proteins (ACSL4, GPX4, SLC7A11) in AR42J cells. **A**–**F** used GAPDH as the reference protein. *P < 0.05, **P < 0.01, ***P < 0.001, ns. not significant. *n* = 6 per group.

Ferroptosis is a form of cell death that depends on the presence of iron and lipid peroxidation in cells. 4-HNE is one of the end products of lipid peroxidation and is often used as an indicator of lipid peroxidation. ACSL4 has been proven to be an important component of executing ferroptosis [30]. SLC7A11 is a member of the system xc-, and ferroptosis occurs when the input of cystine mediated by the system xc- is blocked. GPX4 is an essential ferroptosis regulatory gene. Therefore, inhibiting the activity of antioxidant enzymes, the system xc- and the synthesis of GPX4 can lead to extremely abnormal ROS production and lipid peroxidation, which are high risk factors for inducing ferroptosis [31]. We found that pancreatic injury was aggravated in SIRT4_KO mice with SAP induced by L-Arg when compared to the control group. We also found that 4-HNE expression in tissues was significantly increased (Fig. 6A, Fig. S2E), levels of ACSL4 were increased, and the expression levels of SLC7A11 and GPX4 were decreased (Fig. 6C, Supplementary Fig. 6C). In vitro, AR42J cells showed similar results following the knockdown of SIRT4 (Fig. 6E, Supplementary Fig. 6E). Furthermore, the expression levels of 4-NHE and ACSL4 proteins in tissues decreased, while the expression levels of SLC7A11 and GPX4 proteins increased (Fig. 6B, D, Fig. S2F, Supplementary Fig. 6D, S2F) in AAV_SIRT4 mice with SAP. These results were confirmed in vitro (Fig. 6F, Supplementary Fig. 6F). Based on these data, we proved that SIRT4 suppressed the ferroptosis induced by L-Arg.

### SIRT4 mitigated SAP by suppressing ferroptosis

**Fig. 7:**
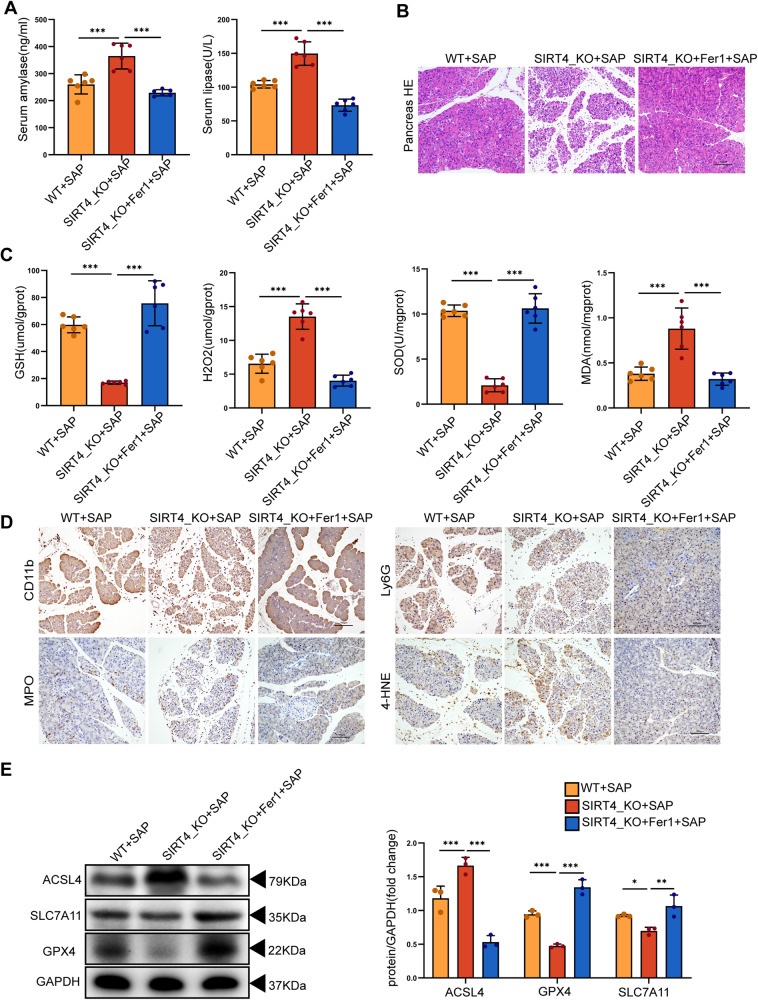
SIRT4 mitigated SAP by suppressing ferroptosis. **A** Fer-1 decreased the serum amylase level and lipase activity of SAP mice induced by L-Arg. **B** Representative HE staining images of pancreas. Scale bar = 100 µm. **C** The levels of oxidative stress (GSH, H_2_O_2_, SOD, MDA) in the pancreas of mice. **D** Representative IHC staining of CD11b, MPO, Ly6G and 4-HNE in the pancreas of mice in different groups. Scale bar = 100 µm. **E** The protein expression level of ferroptosis-related proteins (ACSL4, GPX4, SLC7A11) in the pancreas of mice in different groups. WT + SAP group, SIRT4_KO + SAP group, SIRT4_KO+Fer-1 + SAP group. E used GAPDH as the reference protein. *P < 0.05, **P < 0.01, ***P < 0.001. *n* = 6 per group.

To further investigate the role of ferroptosis in SAP induced by L-Arg, we pretreated mice with the ferroptosis inhibitor ferrostatin-1 (Fer-1, 10 mg/kg). We first verified the inhibitory effect of Fer-1 on the expression of ferroptosis-associated proteins and found that Fer-1 pretreatment reduced trypsinogen activity, as shown in Supplementary Fig. 1E. We further found that compared to mice treated with L-Arg only, those that were pre-treated with Fer-1 showed reduced levels of serum amylase and lipase activity (Fig. 7A); furthermore, histological analysis showed that the extent of pancreatic necrosis was significantly improved (Fig. 7B, Fig. S3A). In addition, we detected the levels of serum inflammatory factors in mice (Fig. S3B), and the expression levels of Ly6G, CD11b and MPO in pancreatic tissue by IHC (Fig. 7D, Fig. S2G). We found that Fer-1 alleviated pancreatic injury and inflammation. Next, we evaluated the main characteristics of ferroptosis, including oxidative stress, lipid peroxidation markers, and the expression of ferroptosis markers. In contrast, Fer-1 significantly reduced H_2_O_2_ and MDA levels, while increasing GSH and SOD levels (Fig. 7C). In addition, 4-HNE (a secondary product of lipid peroxidation) level was significantly increased in the pancreatic tissue injured by L-Arg only, but it was barely detectable in the pancreas of mice treated with Fer-1 (Fig. 7D, Fig. S2G). We detected the expression levels of GPX4, SLC7A11 and ACSL4 in the pancreas by Western blot. We found that the expression levels of GPX4 and SLC7A11 were reduced while those of ACSL4 were increased after L-Arg injection. However, in the Fer-1 treatment group, the expression levels of GPX4 and SLC7A11 were both up-regulated, while ACSL4 was down-inhibited (Fig. 7E, Supplementary Fig. 7E).

In addition, we evaluated the histopathology of the lungs and kidneys in experimental mice. Following L-Arg treatment, the lung tissue showed edema and hemorrhage while the kidneys showed expansion and necrosis of the renal tubules after L-Arg treatment. However, this pathological damage was significantly reduced in the Fer-1 treatment group (Fig. S3C, D). Collectively, these findings indicate that L-Arg-induced SAP was associated with severe lipid peroxidation and ferroptosis. These effects were particularly serious in SIRT4_KO mice; the ferroptosis inhibitor Fer-1 showed a good preventive effect and significantly reduced the severity of SAP. Collectively, these data show that SIRT4 may mitigate SAP by suppressing ferroptosis in mice.

### The regulatory effect of SIRT4 on ferroptosis in SAP depends on the HIF-1α/HO-1 pathway

**Fig. 8:**
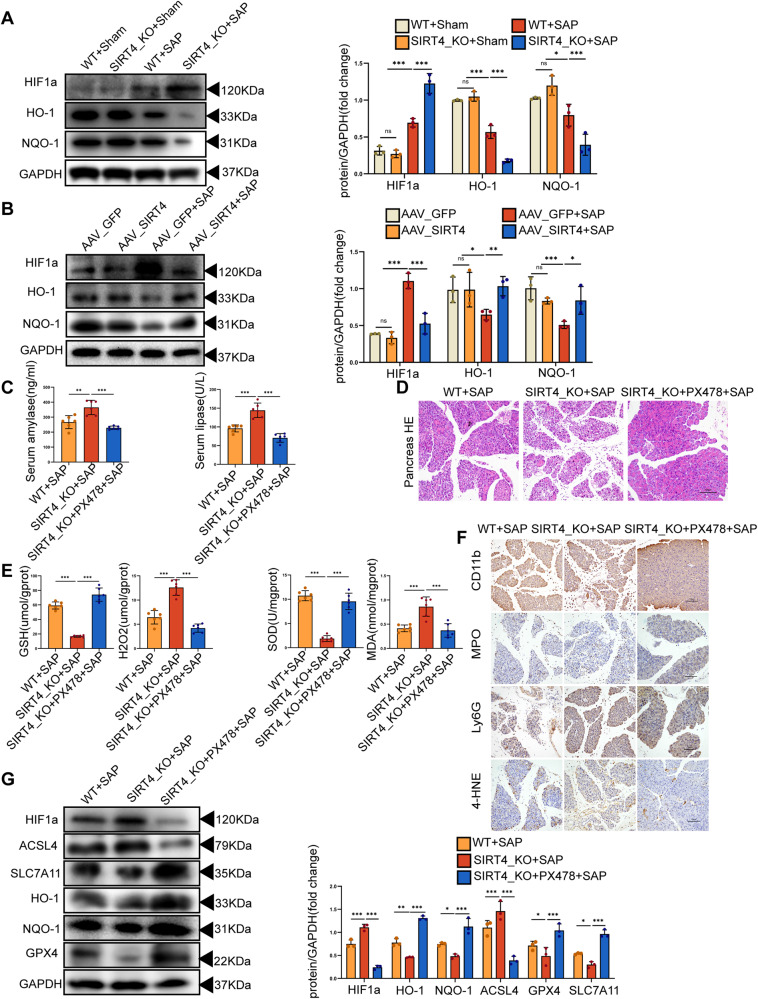
The regulatory effect of SIRT4 on ferroptosis in SAP depends on the HIF-1α/HO-1 pathway. **A**–**B** The protein expression levels of HIF-1α, HO-1 and NQO-1 in the pancreas of mice. **C** Serum amylase level and lipase activity of mice in different groups after using HIF-1α inhibitor PX478 (100 mg/kg). **D** Representative HE staining images of mice pancreas in different groups after using HIF-1α inhibitor PX478 (100 mg/kg). Scale bar = 100 µm. **E** The levels of oxidative stress factors (GSH, H_2_O_2_, SOD, MDA) in the pancreas of mice in different groups after using HIF-1α inhibitor PX478 (100 mg/kg). **F** Representative IHC staining of CD11b, MPO, Ly6G and 4-HNE in the pancreas of mice in different groups after using HIF-1α inhibitor PX478(100 mg/kg). Scale bar = 100 µm. **G** The protein expression level of HIF-1α, HO-1, NQO-1, ACSL, GPX4 and SLC7A11 in the pancreas of mice in different groups after using HIF-1α inhibitor PX478 (100 mg/kg). WT + SAP group, SIRT4_KO + SAP group, SIRT4_KO + PX478 + SAP group. **A**–**G** used GAPDH as the reference protein. *P < 0.05, **P < 0.01, ***P < 0.001, ns. not significant. *n* = 6 per group.

HIF-1α nuclear translocation occurs when cells are under hypoxic conditions and participates in the activation of hypoxia-survival genes. To investigate the role of HIF-1α in SAP, we detected the expression levels of HIF-1α in the pancreatic tissue of mice by Western blot. The expression levels of HIF-1α in the pancreas of control mice were extremely low but increased after L-Arg stimulation; these levels increased in SIRT4_ KO mice following stimulation by L-Arg. We also detected the expression levels of the antioxidant proteins HO-1 and NQO-1 and observed an opposite trend as that shown for HIF-1α (Fig. 8A, Supplementary Fig. 8A). Following L-Arg stimulation, AAV_SIRT4 mice exhibited a lower level of HIF-1α than control group (Fig. 8B, Supplementary Fig. 8B). In vitro, we investigated HIF-1α levels in response to the knockdown or overexpression of SIRT4 in AR42J cells. These results were consistent with those arising from in vivo experiments (Fig. S4A, B, Supplementary Fig. S4A, B). Next, we used the HIF-1α inhibitor PX478 (100 mg/kg) to further investigate the role of HIF-1α in SAP. We found that PX478 significantly reduced the severity of SAP induced by L-Arg, reduced the amylase level and lipase activity, alleviated pancreatic injury (Fig. 8D, S4C), decreased the level of serum inflammatory factors (Fig. S4D), and reduced inflammatory cell infiltration (Fig. 8F). In addition, the levels of MDA and H_2_O_2_ were reduced by PX478 while the levels of GSH and SOD were increased (Fig. 8E). The expression levels of HIF-1α, ACSL4 and 4-HNE were down-regulated after applying PX478, while the expression levels of GPX4, SLC7A11, HO-1 and NQO-1increased (Fig. 8G, F, Supplementary Fig. 8G). Similarly, PX478 also exhibited significant therapeutic effects on the pathology of lung and kidney tissues in experimental mice (Fig. S4E, F). Collectively, these results showed that HIF-1α played a key role in SAP and that its inhibitor (PX478) exerted therapeutic effects on L-Arg-induced SAP. The expression levels of HIF-1α were reduced following the overexpression of SIRT4; similarly, both lipid peroxidation and ferroptosis were significantly reduced. Therefore, SIRT4 may inhibit HIF-1α and inhibit lipid peroxidation and ferroptosis, thereby playing a protective role in SAP.

## Discussion

The etiology of AP is complex and the pathogenesis underlying this condition -involves a range of factors and has yet to be fully clarified [32]. Inflammatory reactions and oxidative stress play a key role in SAP. SIRT4 plays a key role in the regulation of inflammation and oxidative stress. By searching a public database, we found that the expression of SIRT4 in AP patients was down-regulated when compared with that in healthy subjects. However, it is unclear whether SIRT4 can protect SAP.

During AP, both macrophages and neutrophils aggregate in the pancreas and release inflammatory cytokines. When this condition progresses to SAP, the pancreas undergoes severe necrosis; the resulting inflammatory reaction cascade can then affect multiple organs in the body, leading to tissue damage and dysfunction [33]. In the present study, we found that severe pancreatic injury occurred in mice in L-Arg-induced SAP. HE and IHC analysis revealed varying degrees of edema, necrosis, and inflammatory cell infiltration in pancreatic tissue. Moreover, the levels of serum inflammatory factors (IL-6, IL-1β, TNF-α and MCP-1) were increased significantly in experimental mice. There is a dynamic balance between oxidation and antioxidation in normal pancreatic tissue [7]. Many studies have shown that oxidative stress is an early event of AP [34]. The direct effect of oxidative stress, represented by an increase in ROS, on acini leads to the damage being incurred by lipid, protein, and DNA. Furthermore, ROS can also increase the activation of inflammatory signals; the interaction between ROS and other signaling pathways will amplify the inflammatory cascade reaction [35]. Inflammatory reactions and oxidative stress interact to aggravate pancreatic injury. Ferroptosis is a form of cell death caused by severe lipid peroxidation and depends on the production of ROS and the accumulation of iron. The main causes of ferroptosis are GSH depletion and the subsequent inactivation of GPX4 [31]. In a previous study, Li et al. proved that the activation of GPX4 can reduce the levels of ROS in cells and the inflammatory response mediated by ROS [36]. Ferroptosis is involved in other pathological processes, including myocardial disease, liver injury and kidney injury [37]. It has been confirmed that AP is associated with excessive levels of iron in the pancreas and a consequential increase in the levels of ROS [38]. In a previous study, Ma et al. found that experimental SAP in rats can cause related kidney damage, iron accumulation, lipid peroxidation and the upregulation of ferroptosis-related proteins in the pancreas [39]. In another study, Fan et al. proved that pyroptosis and ferroptosis play a key role in AP [12]. In the present study, we confirmed the role of oxidative stress and ferroptosis in SAP. We also found that the levels of GSH and SOD decreased in SAP mice and AR42J cells induced by L-Arg, while the levels of MDA and H_2_O_2_ increased significantly. The expression levels of ACSL4 protein were increased, while the expression levels of GPX4 and SLC7A11 proteins were decreased in SAP. In conclusion, our study demonstrated that SAP is associated with severe inflammation, oxidative stress and ferroptosis.

SIRT4 plays an important role in many diseases due to extensive enzymic activity and range of substrates [17, 40]. SIRT4 inhibits inflammation by inhibiting mast cell degranulation [41]. In a previous study, Dai et al. found that SIRT4 protein treatment can inhibit the inflammatory response and oxidative stress associated with osteoarthritis [42]. In another study, Shi et al. reported that the overexpression of SIRT4 reduced inflammation and the production of ROS [43]. SIRT4 has also been shown to reduce the oxidative stress caused by heat stress by improving mitochondrial function [44]. Collectively, these results indicate that SIRT4 has significant potential for regulating inflammation and oxidative stress. In this study, compared with normal mice, SIRT4_KO mice with SAP showed more severe inflammatory reactions and oxidative stress. SIRT4 deletion significantly increased the levels of proinflammatory factors (IL-6, IL-1β, TNF-α and MCP-1), increased the infiltration of inflammatory cells in pancreatic tissue, reduced the levels of GSH and SOD, and increased the levels of MDA and H_2_O_2_ in mice with SAP. We found that the expression of ACSL4 protein was increased in the pancreas after SIRT4 deletion, while the expression of GPX4 and SLC7A11 proteins were reduced by western blot analysis. AAV_SIRT4 mice showed mild SAP symptoms, inflammatory and oxidative stress. SIRT4 reversed the reduction of GPX4 and SLC7A11 levels induced by L-Arg. The protective effect of SIRT4 on AR42J cells stimulated by L-Arg was also confirmed in vitro. In order to further investigate the regulatory effect of SIRT4 on ferroptosis in SAP, we used Fer-1 to treat SIRT4_KO mice in SAP. Fer-1, an effective and selective inhibitor of ferroptosis, reduces unstable iron by forming complex with iron and lipid hydroperoxide by alkoxy radical [45]. Our results showed that the levels of inflammatory reaction and oxidative stress in mice were significantly reduced after treatment with Fer-1. Thus, our study confirmed the anti-inflammatory and antioxidant stress effects of SIRT4 in SAP. SIRT4 could protect the pancreas from ferroptosis by reducing the accumulation of intracellular lipid ROS and alleviating oxidative stress injury.

Once initiated, SAP will cause damage to other organs. The common clinical manifestations are shock, acute respiratory failure, and acute kidney injury (AKI). Pancreatic necrosis, bacteremia and the cascade amplification effect of inflammation cause diffuse alveolar injury, a condition referred to as acute lung injury (ALI) [46]. AKI is also a common complication of SAP which often occurs after the failure of other organs. The mechanism underlying AKI is so complex that the interaction of low blood volume, severe inflammatory reaction, and toxic substances released by pancreatic necrosis, may cause serious damage to the kidney. In this study, we performed HE staining analysis on lung and kidney tissues taken from mice after L-Arg-induced SAP. We found that edema and inflammatory infiltration of lung tissue in SIRT4_KO mice were both aggravated when compared with SAP mice. In contrast, AAV_SIRT4 mice experiencing SAP showed reduced levels of lung damage. Similarly, SIRT4 deficiency caused damage to the renal tissue, including tubular necrosis, tubular dilatation, brush border disappearance and remodeling. Therefore, the overexpression of SIRT4 not only alleviated pancreatic injury, but also alleviated SAP-related lung and kidney tissue injury.

HIF-1α is a major regulator that allows mammalian cells to adapt to hypoxia. Proline residues of HIF-1α are known to be hydroxylated under normal oxygen conditions and degraded by the ubiquitin-proteasome pathway. In hypoxia, this degradation pathway is inhibited and HIF-1α exists at steady levels in cells. HIF-1α has been proven to be a key molecule in many hypoxic-ischemic diseases and tumorigenesis [47–50]. Due to the aggregation of immune cells, the inflammatory site creates a poor environment associated with rapid hypoxia, thus leading to the transcription of HIF-1α by immune cells. HIF-1α is the signal transduction center for hypoxia and regulates the expression of related molecules downstream. Lee et al. demonstrated that hypoxia and HIF-1α accumulation occurred during experimental pancreatitis [51]. In another study, Park et al. found that HIF-1α aggravated AP induced by Cerulein(CER) and promoted coagulation and tissue damage in the pancreas [52]. HIF-1α is closely related to ROS, and HIF-1α can be detected with the aggravation of tissue hypoxia and increased levels of ROS. Wu et al. found that hypoxia promoted the release of ROS and mitochondrial fission via HIF-1α [53]. HO-1 plays a protective role with anti-inflammatory, anti-tumor, anti-oxidation, anti-apoptosis, and other effects, by converting heme into antioxidant biliverdin, clearing ROS and inhibiting lipid peroxidation [54, 55]. It was found that HO-1 can reduce ferroptosis in renal proximal tubule cells [56] and that the elimination of HO-1 can aggravate the ferroptosis induced by erastin and sorafenib [57]. According to previous research, HIF-1α can induce the expression of HO-1 and inhibit the production of ROS. However, the application of the HO-1 inhibitor zinc protoporphyrin IX led to increase in the expression of HIF-1α [58]. Therefore, the levels of HIF-1α increased while the expression of HO-1 was downregulated during excessive oxidative stress injury. It has been implicated that the downregulated of HO-1 by HIF-1α may be related to the reduced expression of Nrf2 [59]. In our study, we found that an imbalance between the oxidation and antioxidant systems led to severe lipid peroxidation in L-Arg-induced SAP, with HIF-1α expression up-regulated and HO-1 expression down-regulated. In addition, the absence of SIRT4 caused a significant increase in HIF-1α, while the overexpression of SIRT4 inhibited the expression of HIF-1α. PX478 is an inhibitor of HIF-1α, which can inhibit the expression of HIF-1α under normal and hypoxic conditions. HIF-1α expression decreased and ferroptosis was reversed following treatment with PX478 in mice with SAP. These results suggested that SIRT4 may alleviate SAP by inhibiting ferroptosis and regulating the HIF-1α/HO-1 pathway.

This study has some limitations that need to be considered. First, there are too many complicated mechanisms involved with human SAP to imitate in a mouse model of SAP induced by L-Arg. Therefore, the conclusions of this study need to be further confirmed by future research. Secondly, this study proved that SIRT4 alleviated SAP by regulating HIF-1α. However, whether there is a direct interaction between SIRT4 and HIF-1α needs to be confirmed by further molecular research.

In conclusion, our research identified a novel mechanism by which SIRT4 can protect from SAP and demonstrated that HIF-1α plays a significant role in SAP. SIRT4 was shown to play a protective role in SAP mice by regulating the HIF-1α/HO-1 pathway to inhibit ferroptosis related to oxidative stress.

### Supplementary information

Original data files---western blots

Supplemental materials

Reproducibility checklist

## Data Availability

The datasets generated or analyzed during this study are available from the corresponding authors on reasonable request.
