# Supplementary material for: Sirtuin4 alleviates severe acute pancreatitis by regulating HIF-1α/HO-1 mediated ferroptosis
Source: Cell Death Dis. 2023 Oct 21;14(10):694. doi: 10.1038/s41419-023-06216-x (PMC10590376; doi:10.1038/s41419-023-06216-x)

Supplemental Fig. 1

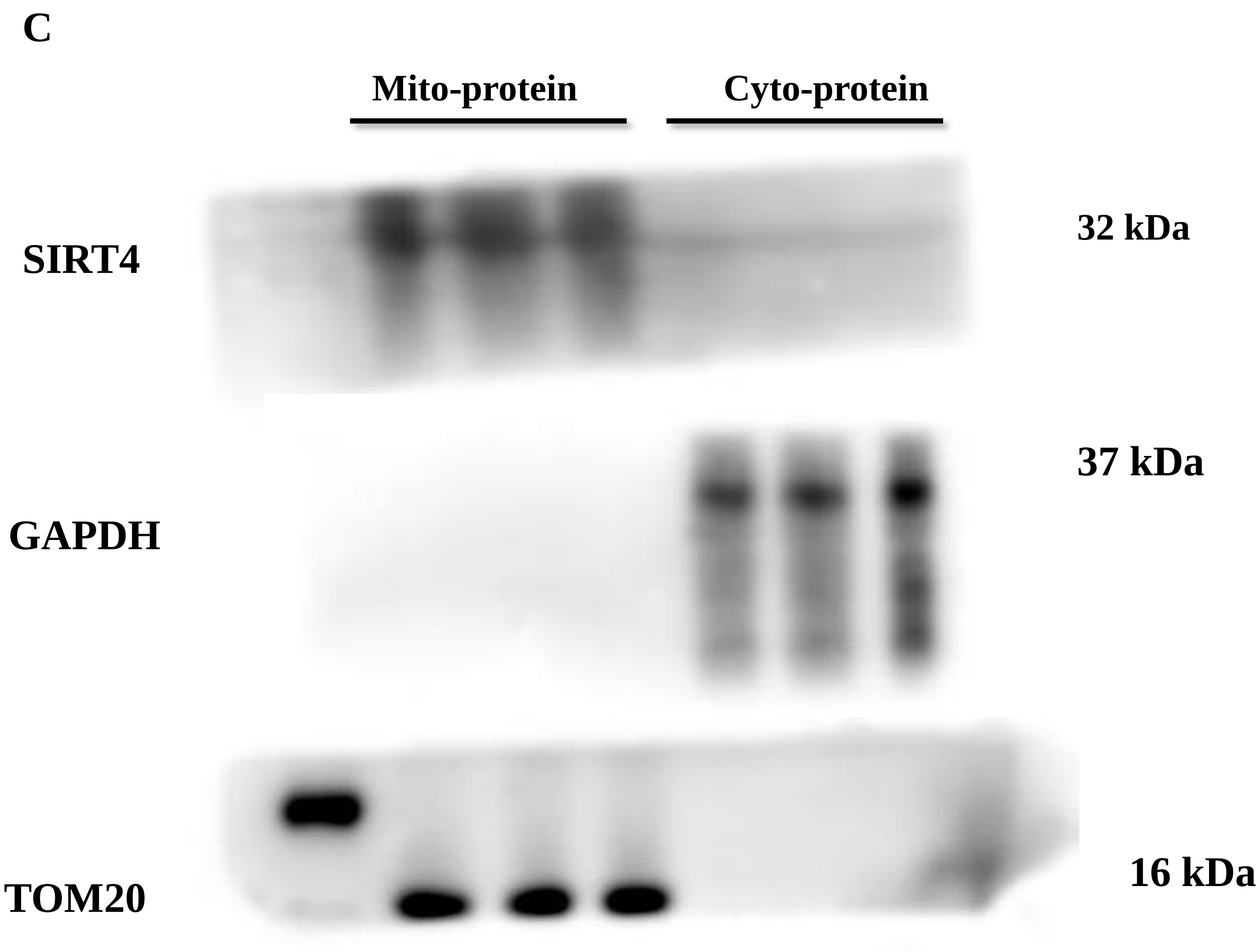

Supplemental Fig. 1

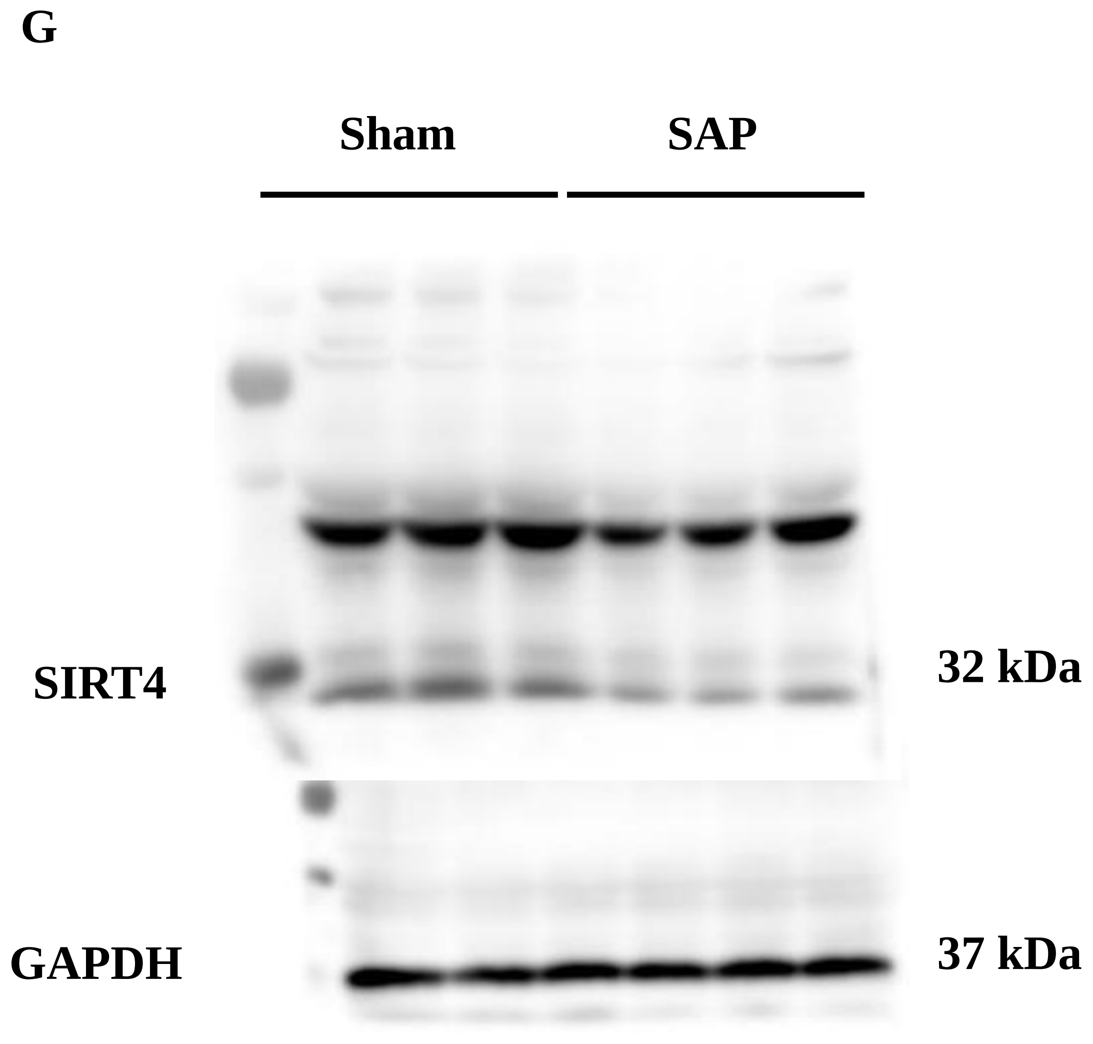

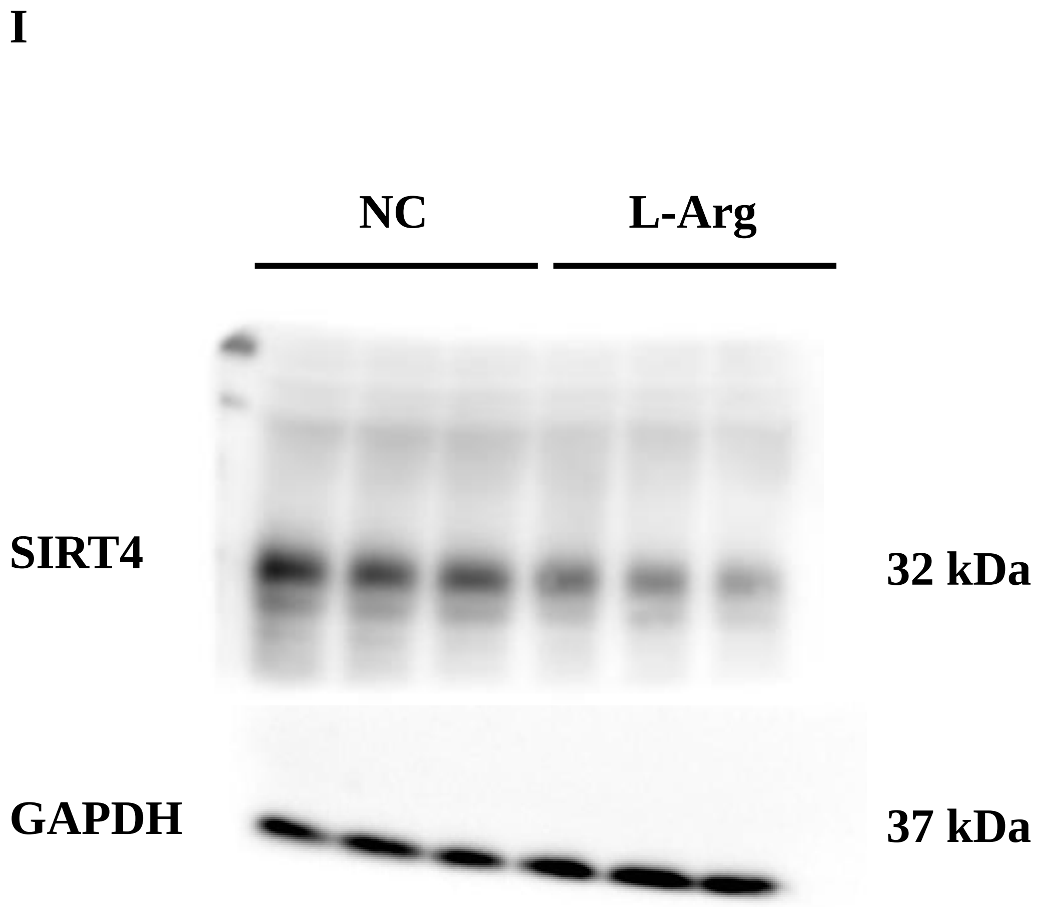

Supplemental Fig. 2

B

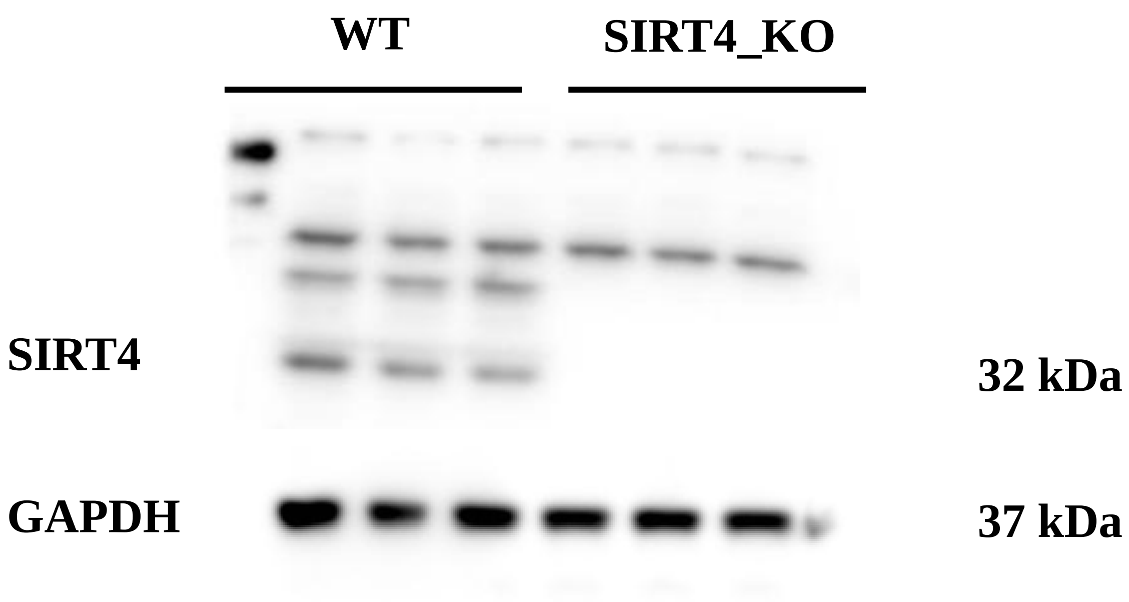

Supplemental Fig. 5

A

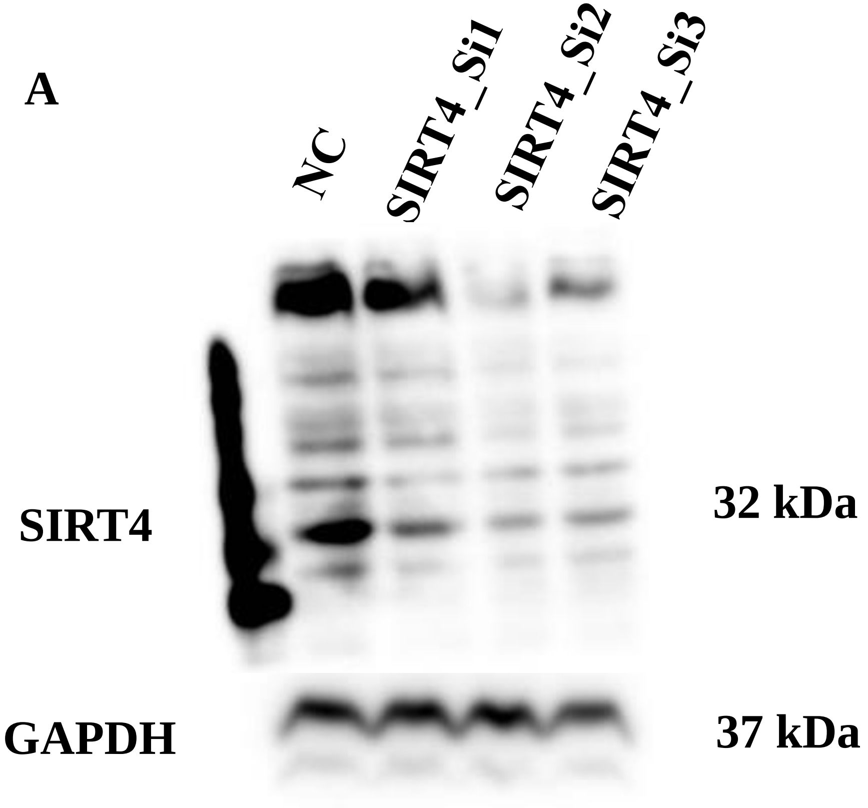

B

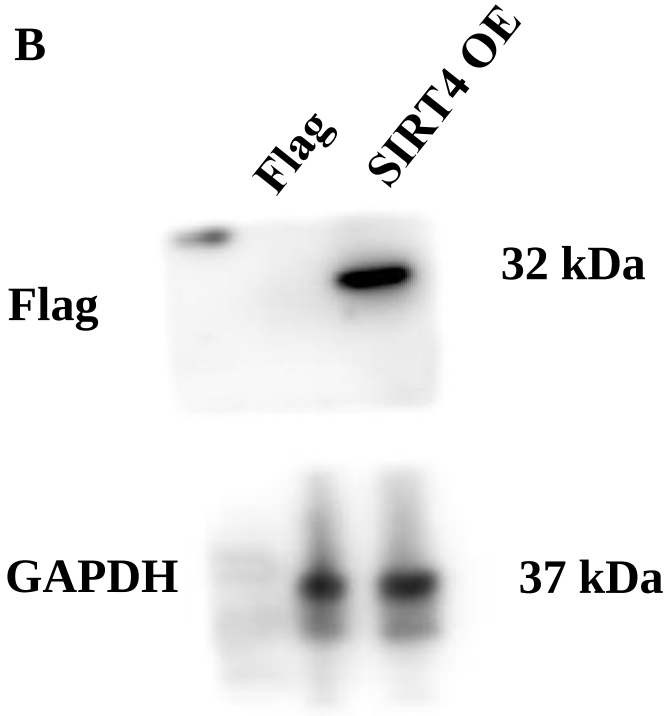

Supplemental Fig. 6

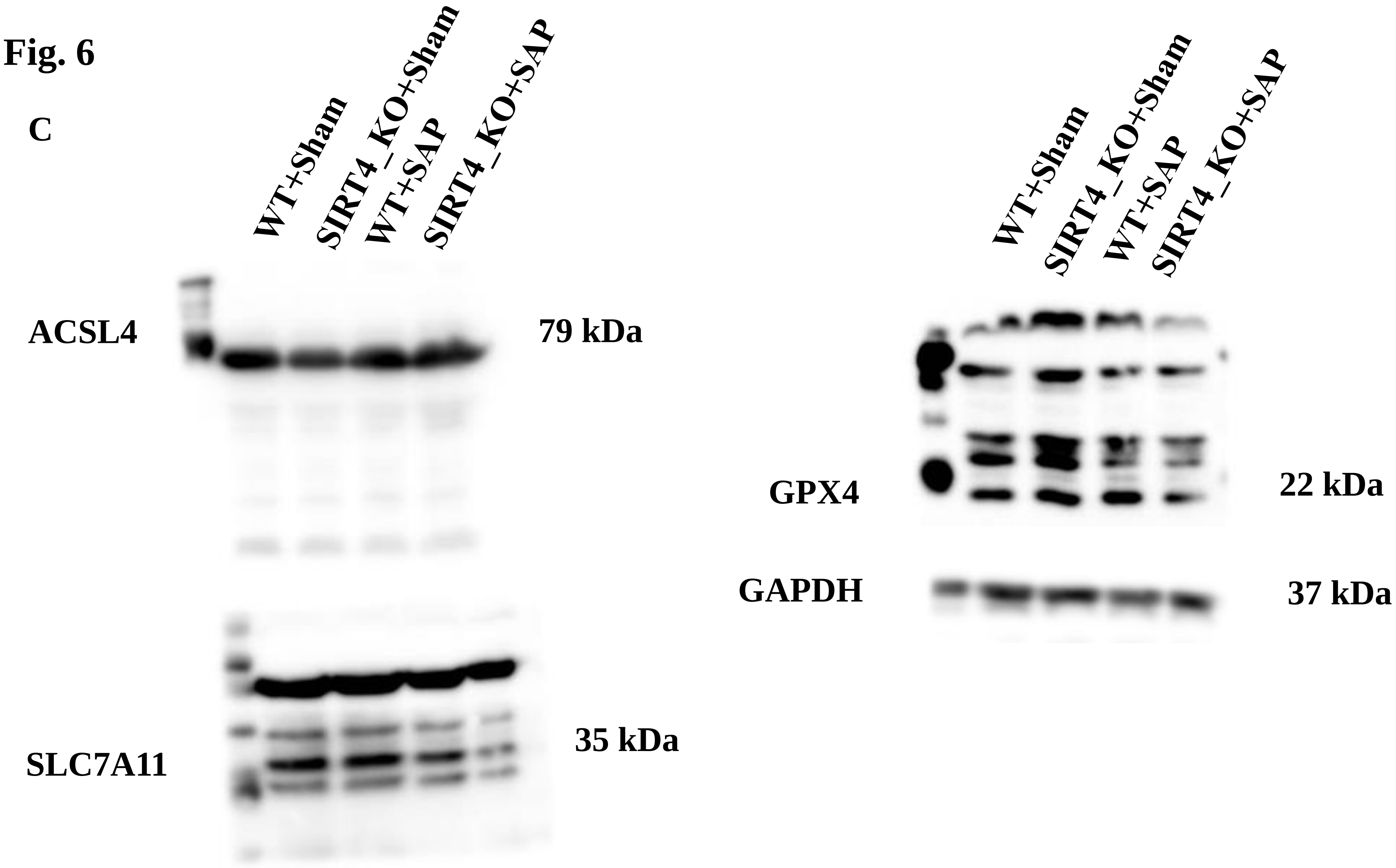

Supplemental Fig. 6

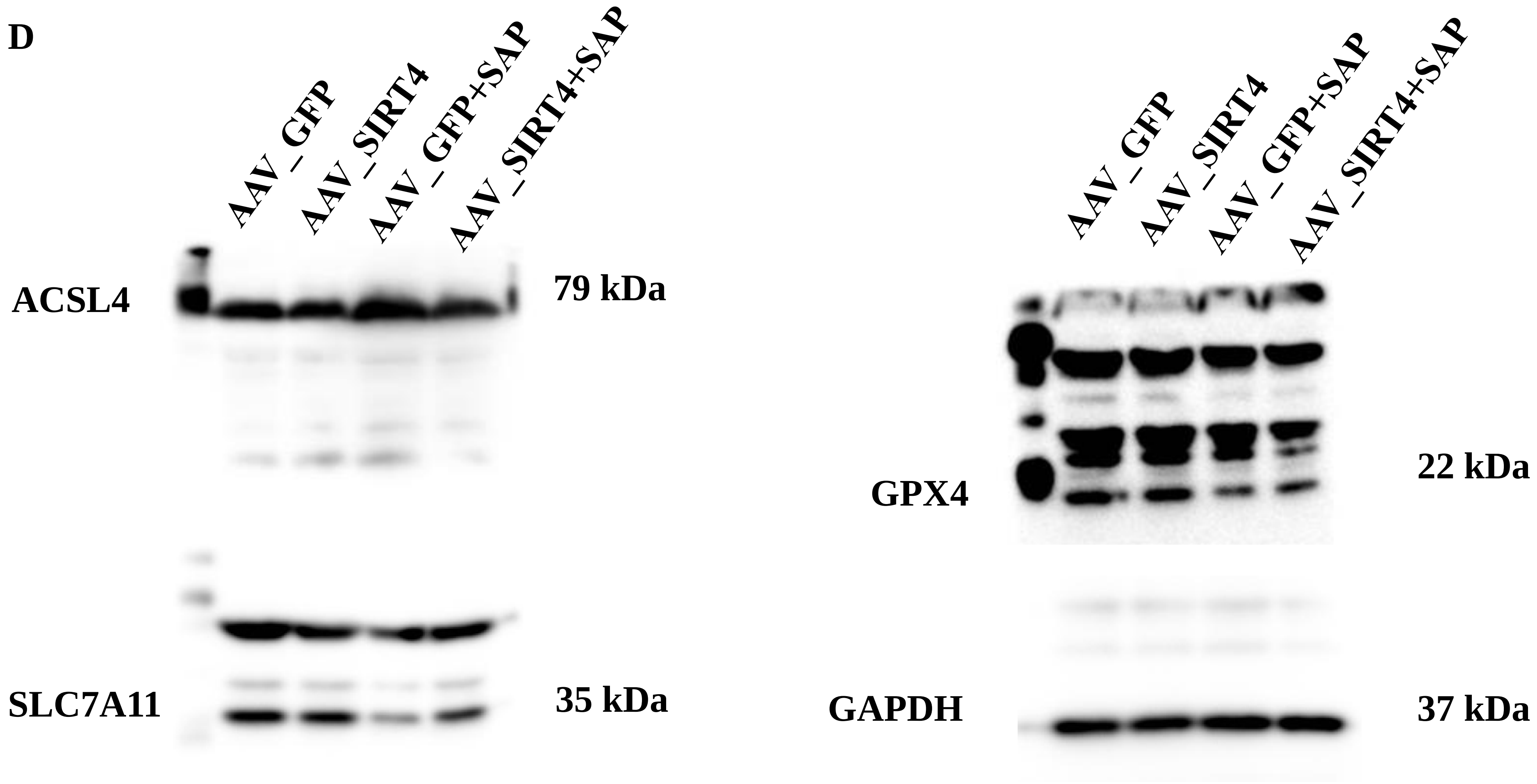

Supplemental Fig. 6

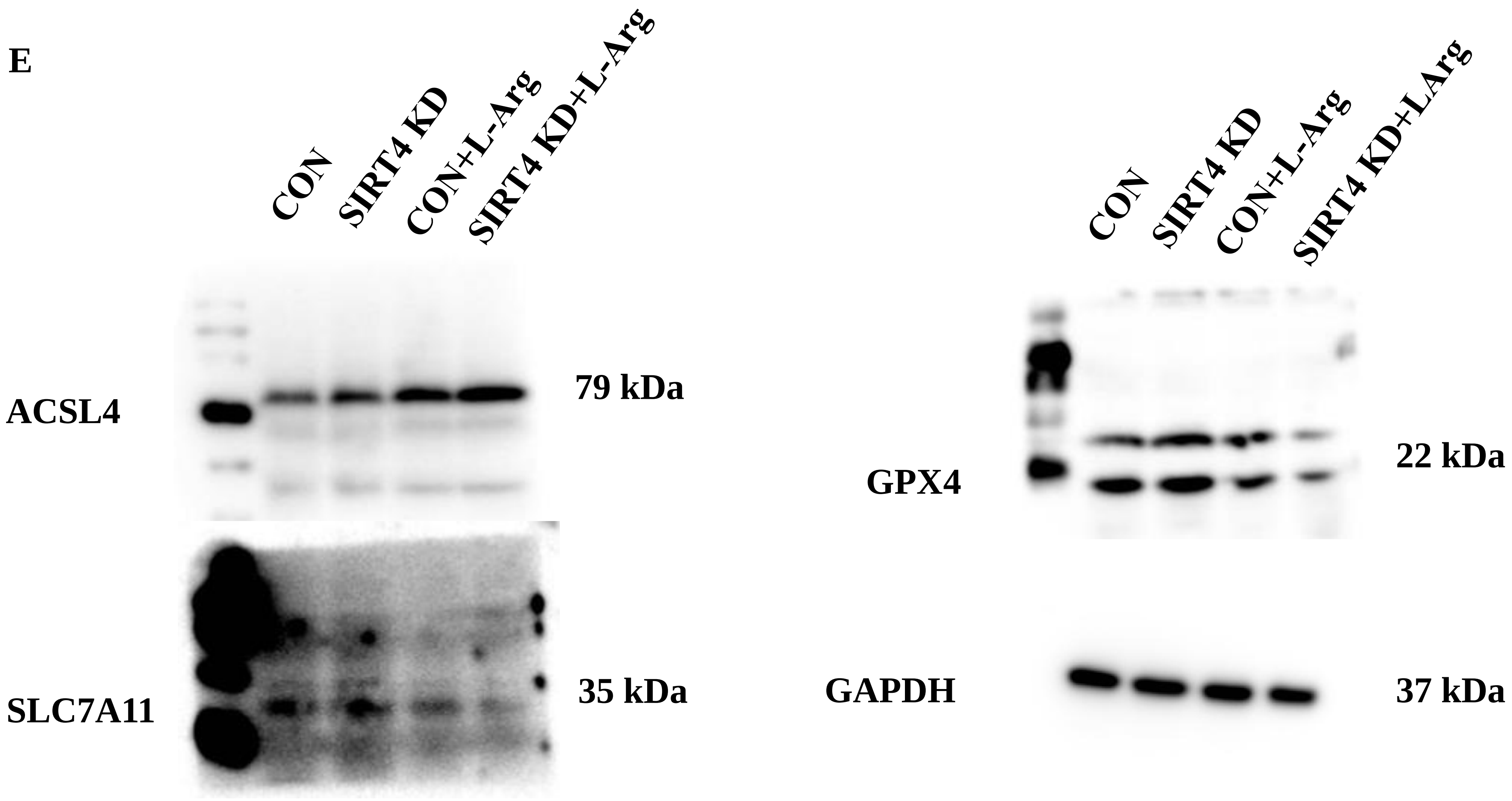

Supplemental Fig. 6

F

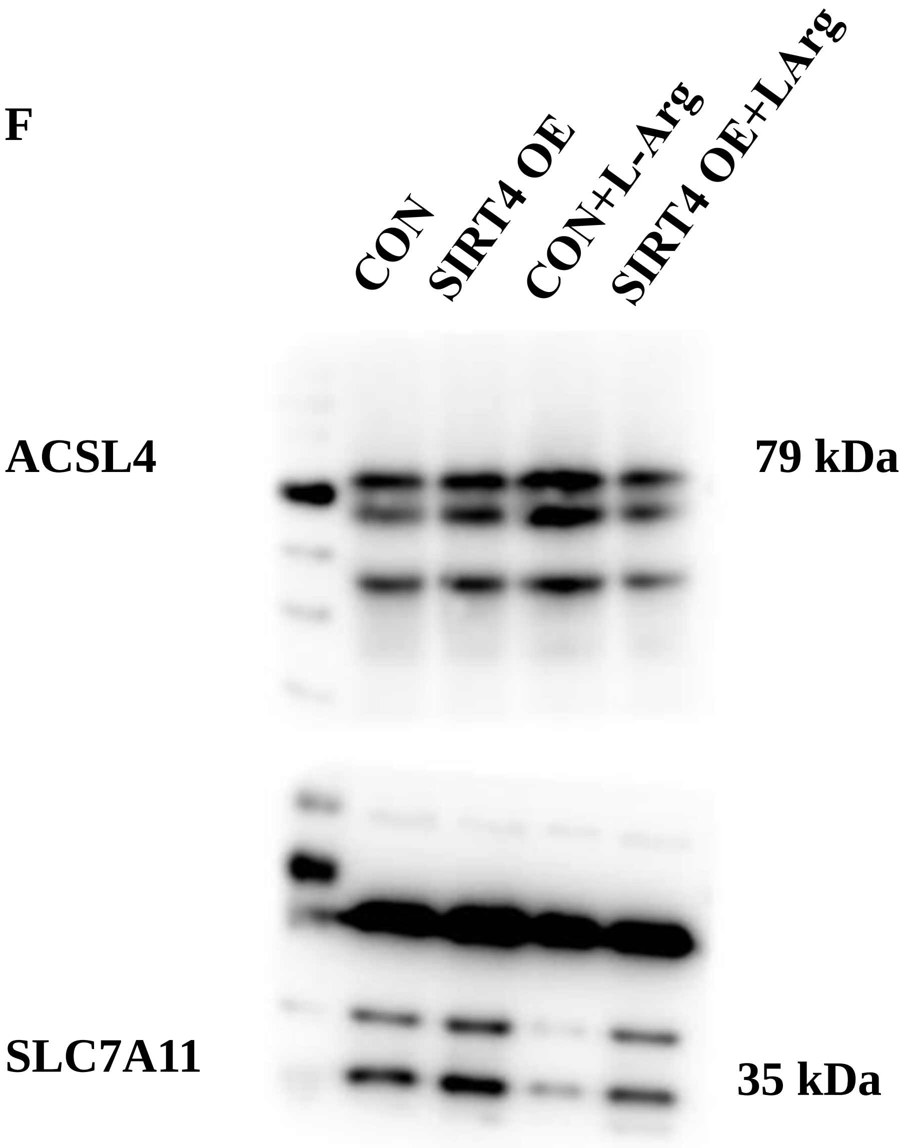

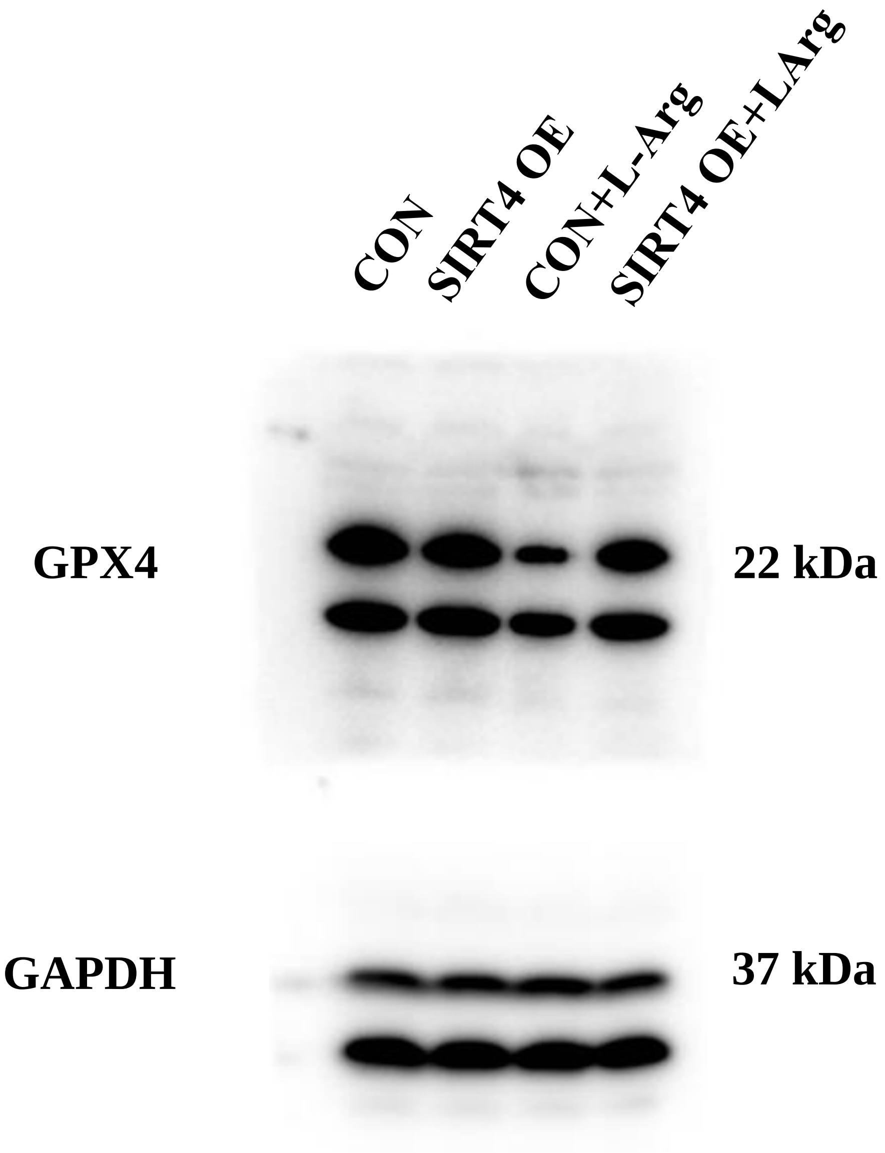

Supplemental Fig. 7

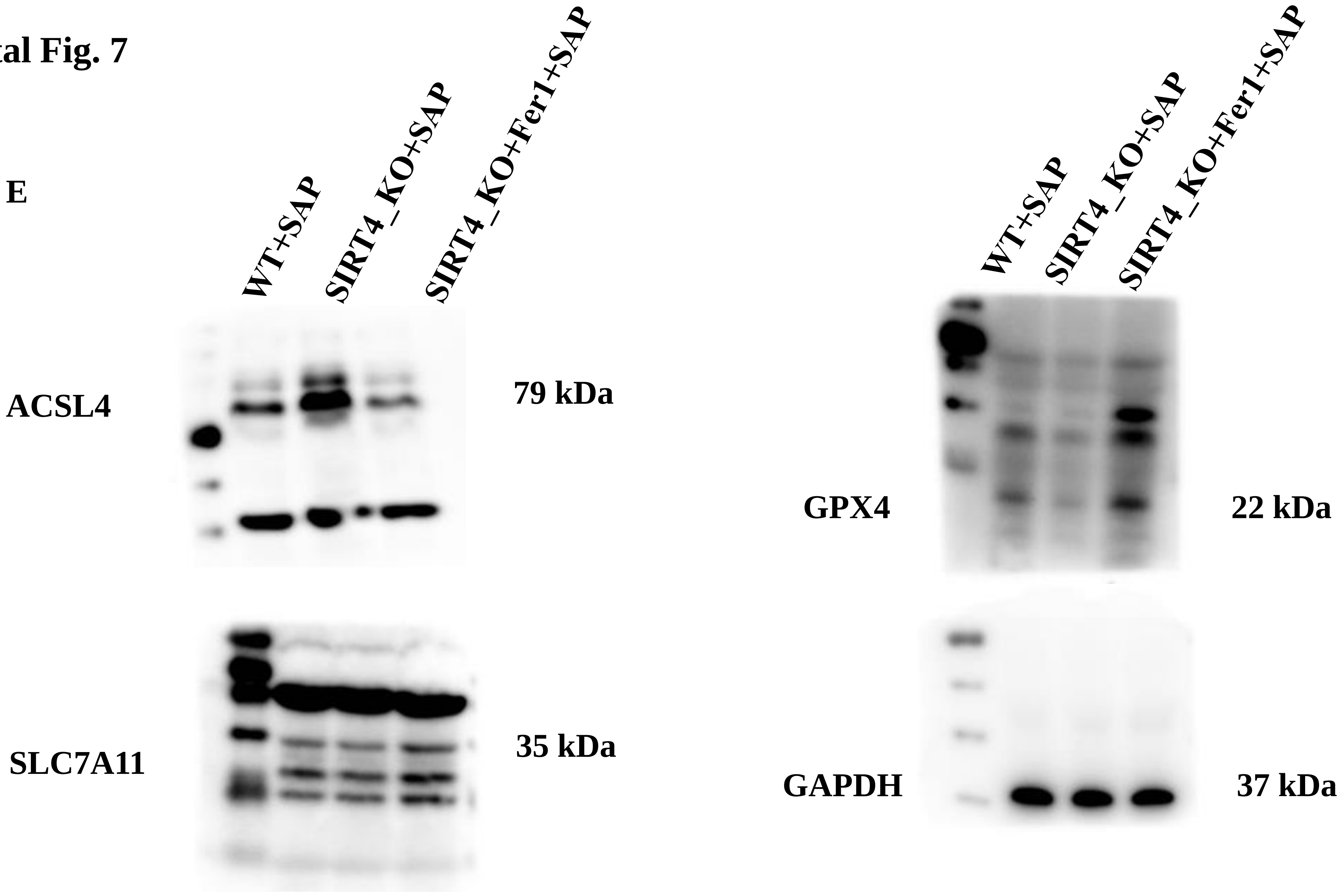

Supplemental Fig. 8

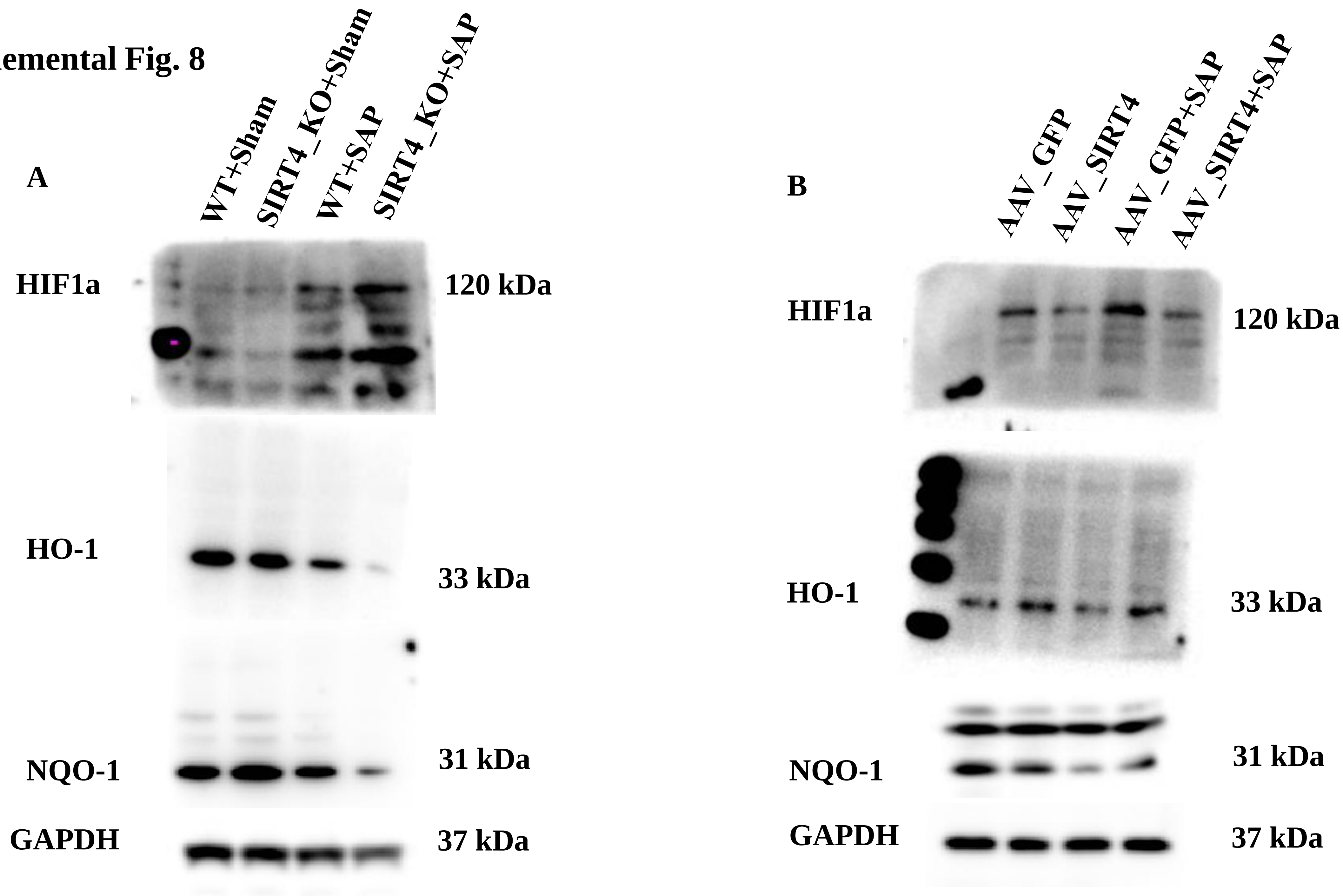

Supplemental Fig. 8

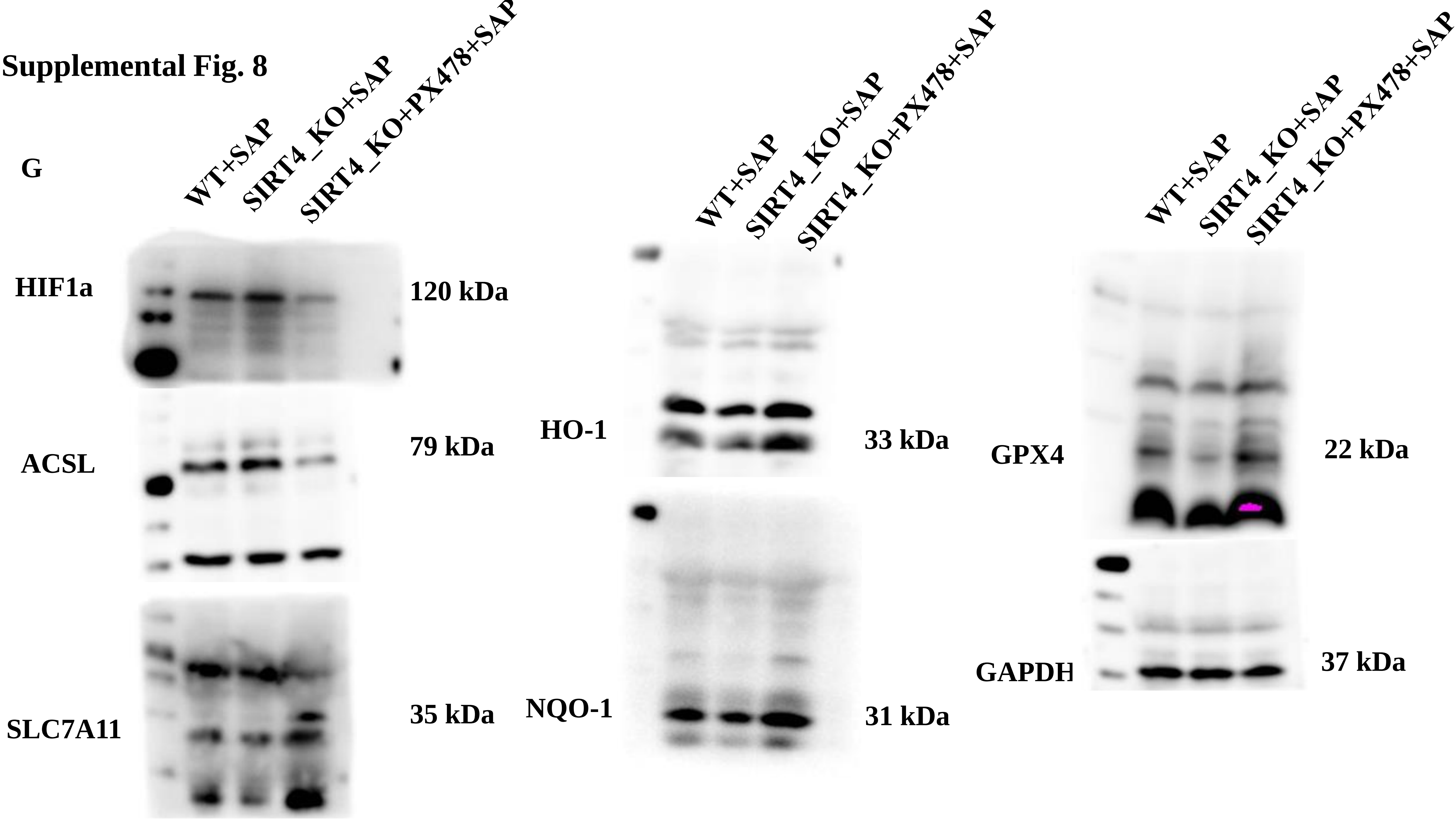

Supplemental Fig. S1

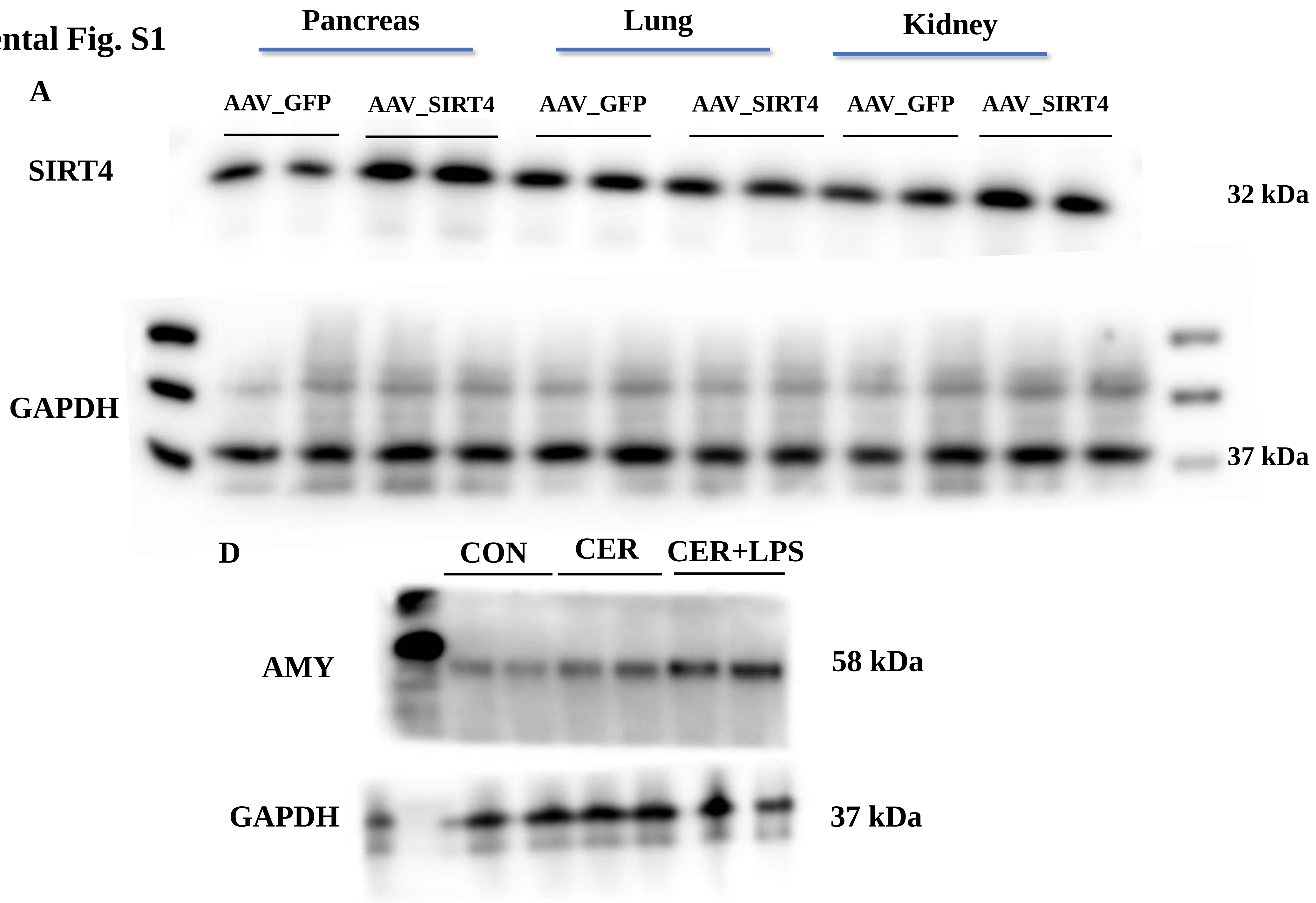

Supplemental Fig. S1

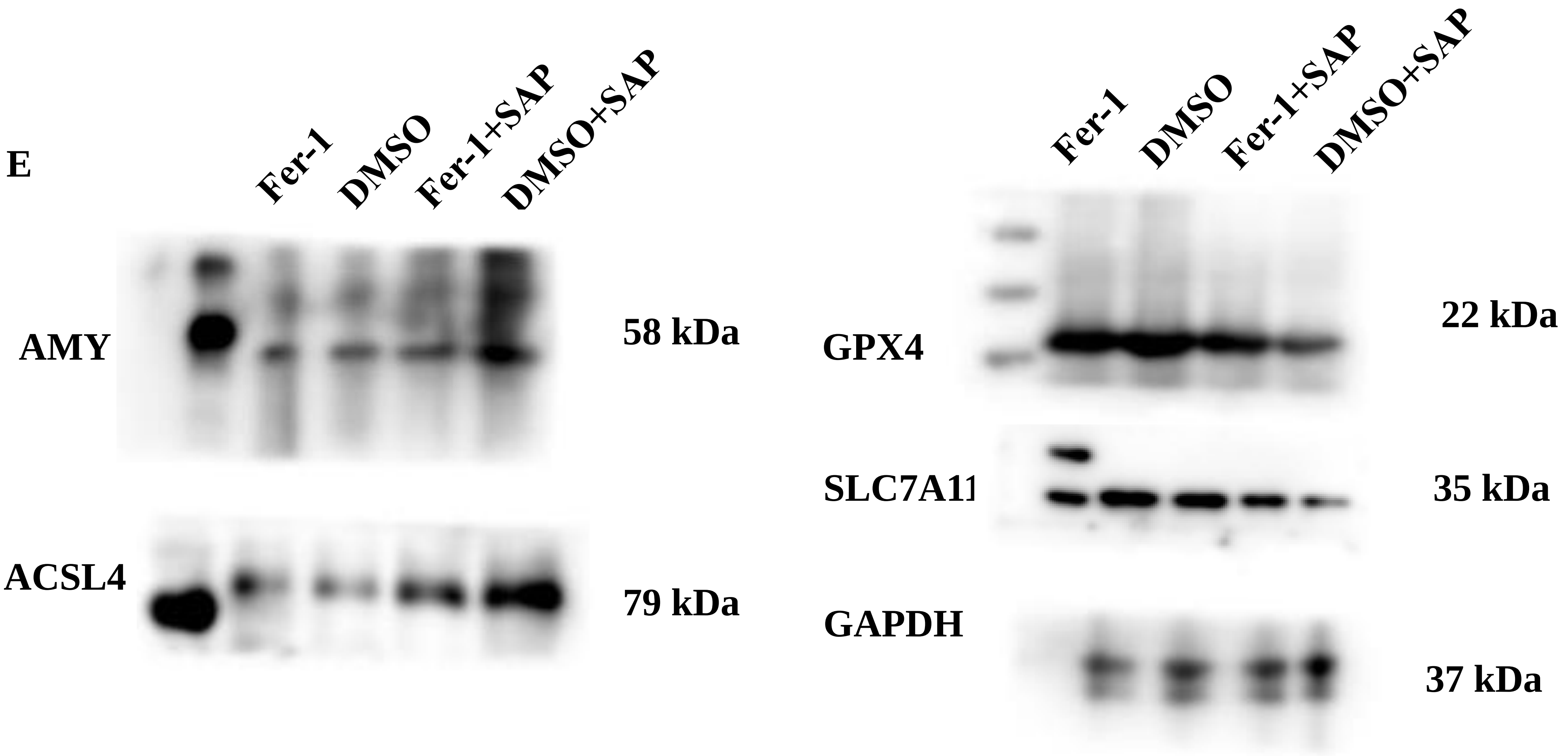

Supplemental Fig. S2

A

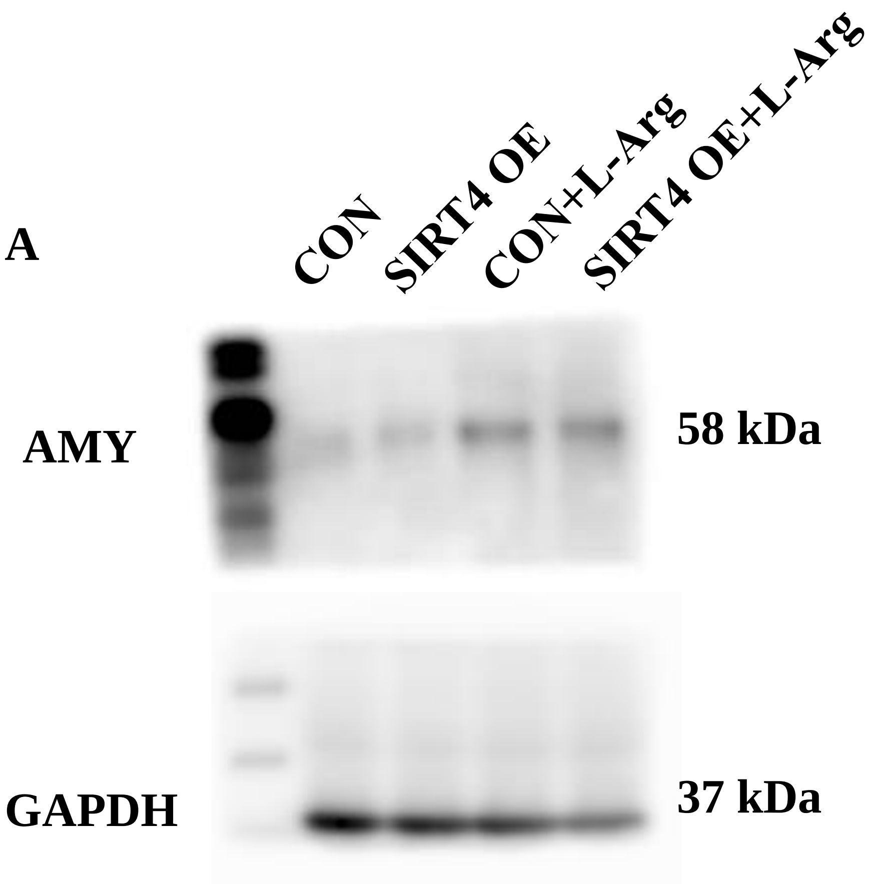

Supplemental Fig. S4

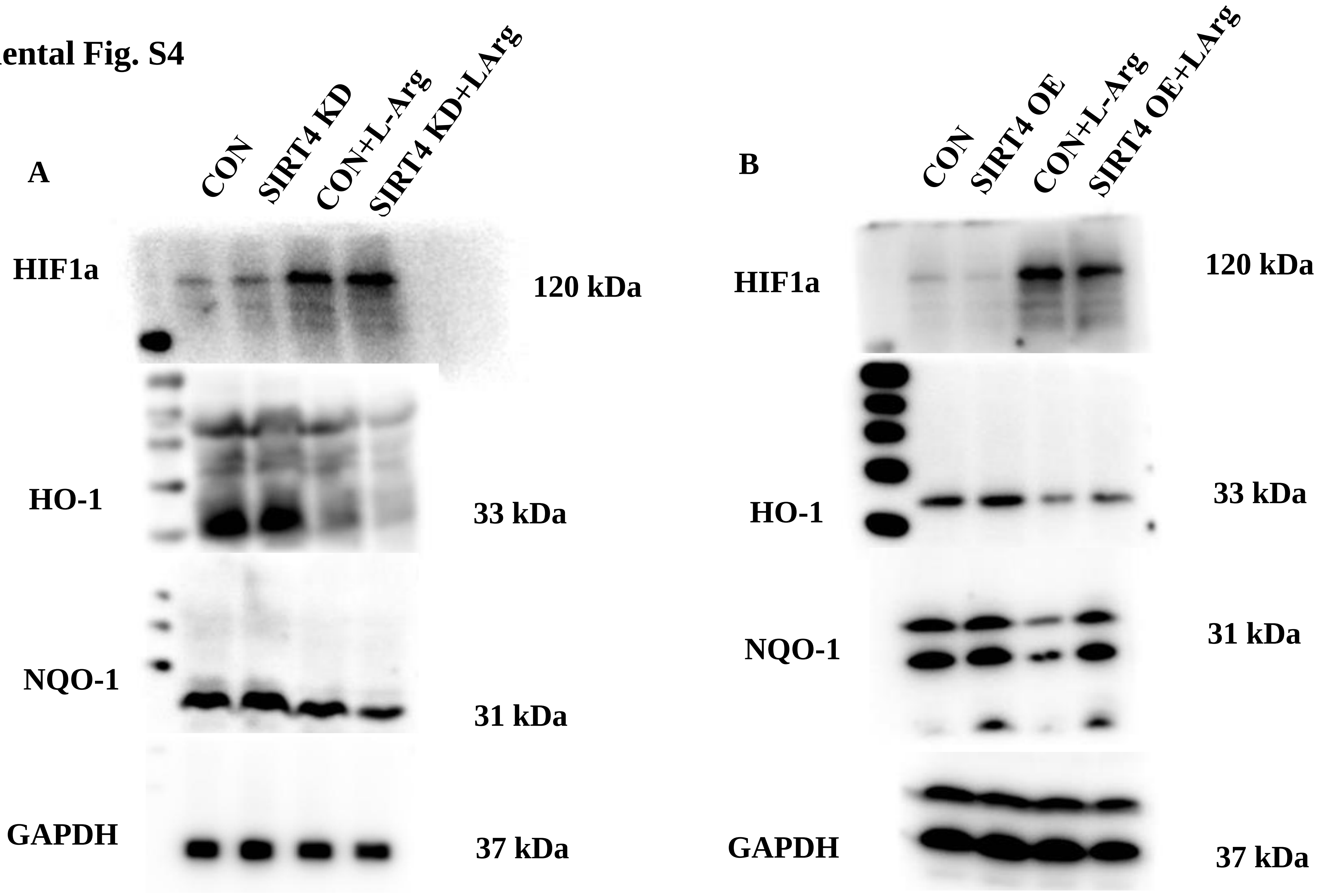

Supplement: Supplementary file 1 — Original data files---western blots [file 41419_2023_6216_MOESM1_ESM.pdf]
