# Supplementary material for: Sirtuin4 alleviates severe acute pancreatitis by regulating HIF-1α/HO-1 mediated ferroptosis
Source: Cell Death Dis. 2023 Oct 21;14(10):694. doi: 10.1038/s41419-023-06216-x (PMC10590376; doi:10.1038/s41419-023-06216-x)
Supplement: Supplementary file 2 — Supplemental materials [file 41419_2023_6216_MOESM2_ESM.pdf]

Supplemental materials

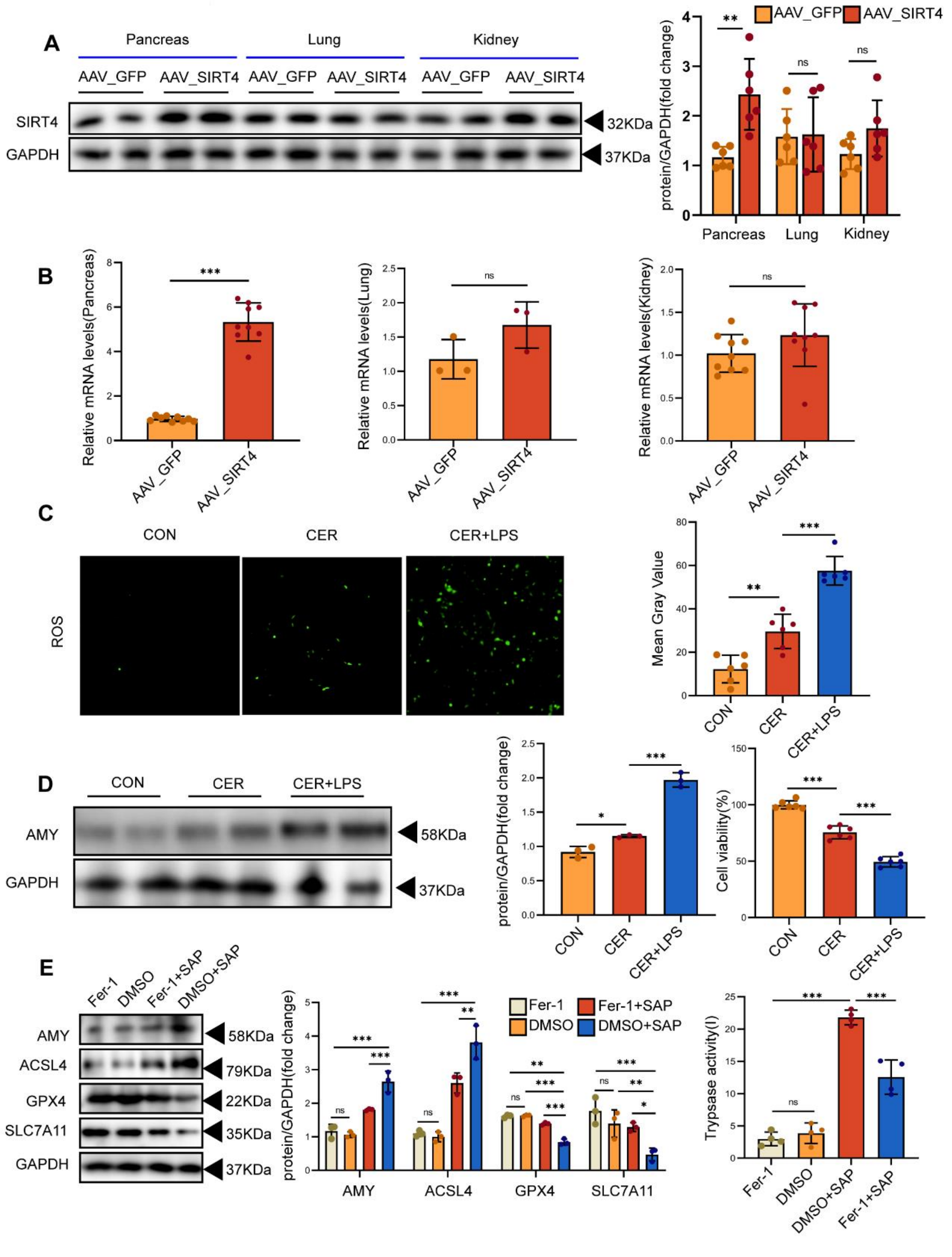

Fig. S1 (A) Expression and quantification of SIRT4 protein in pancreas, lungs, and kidneys after AAV-mediated SIRT4 overexpression. (B) Expression levels of SIRT4 mRNA in the pancreas, lungs, and kidneys following AAV-mediated SIRT4 overexpression. (C) Representative immunofluorescence plots and quantitative analysis of ROS expression in CER and CER+LPS models. (D) Protein expression levels and

quantification of amylase in the CER and CER+LPS models, as well as the expression of cell activity in different experimental models. (E) Pretreatment with Fer-1 affected the expression of ferroptosis-related proteins and reduced amylase expression and trypsase activity. * $P < 0.05$, ** $P < 0.01$, *** $P < 0.001$, ns., not significant. n = 6 per group.

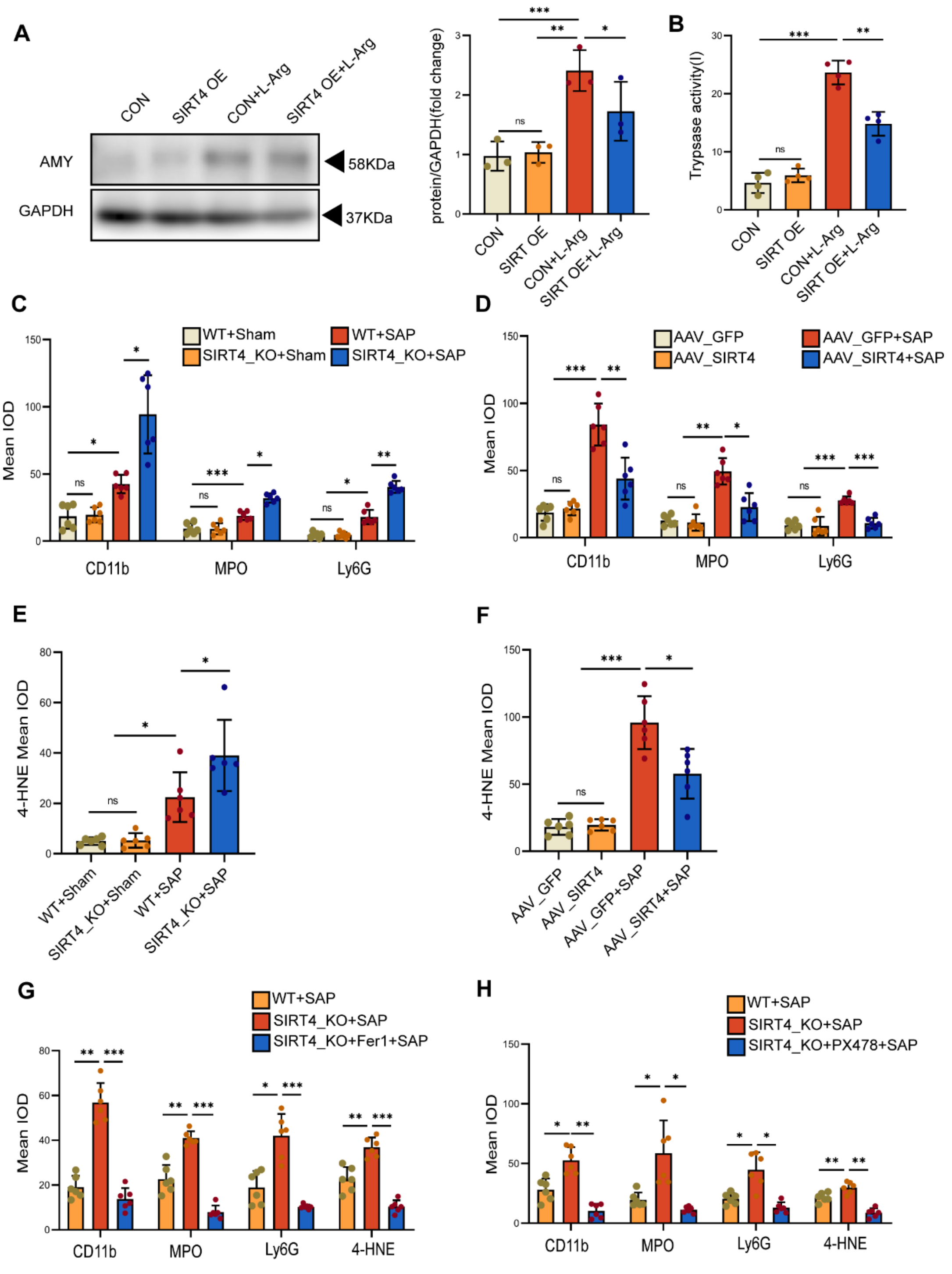

Fig. S2 (A) SIRT4 overexpression reduces amylase secretion after induction of SAP

in AR42J cells. (B) SIRT4 overexpression reduces trypsin activity after induction of SAP in AR42J cells. (C-H) Quantitative analysis plots of IHC images in Fig. 4, Fig. 6, Fig. 7 and Fig. 8 in turn. * P<0.05, **P<0.01, ***P<0.001, ns., not significant. n = 6 per group.

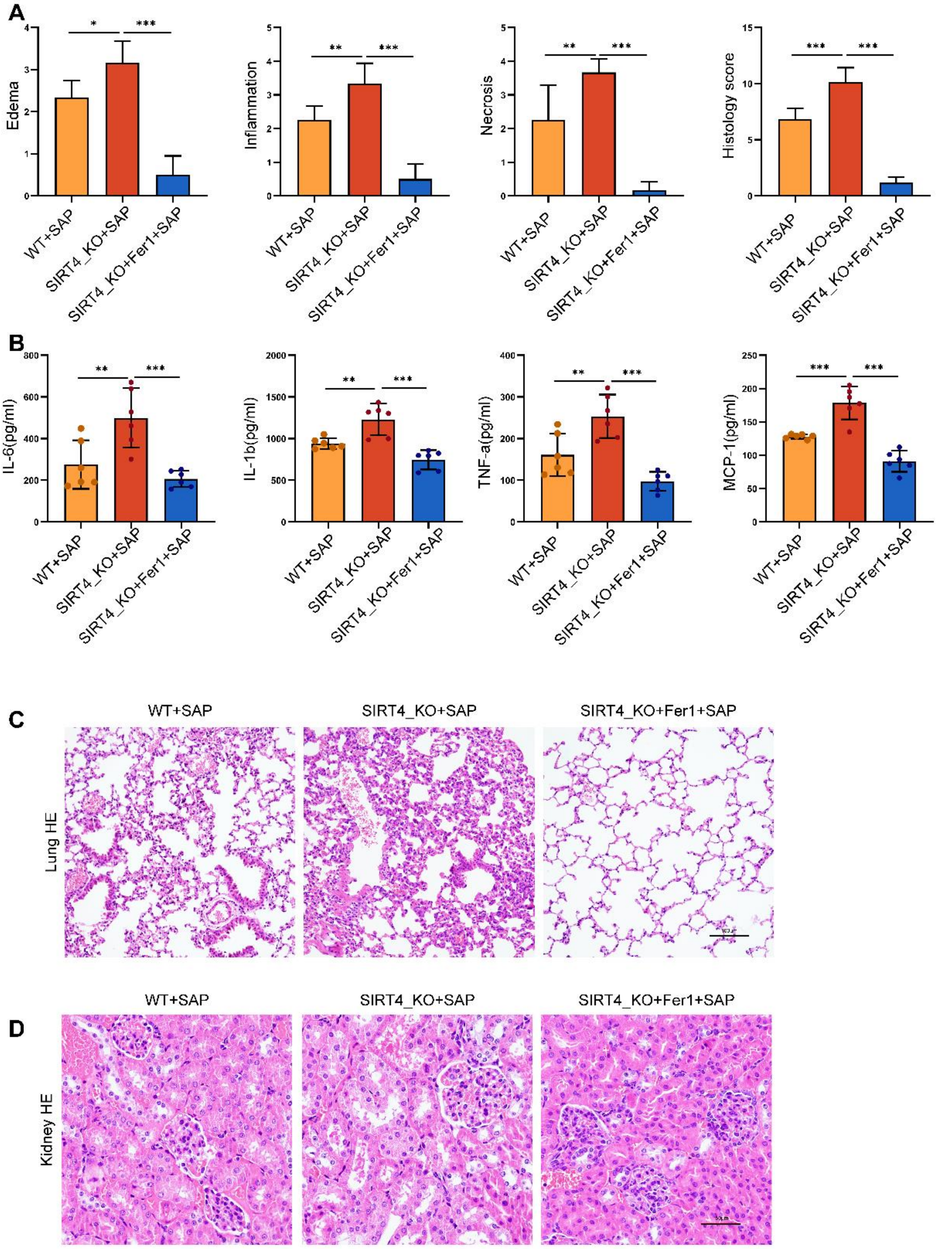

Fig. S3 SIRT4 mitigated SAP by suppressing ferroptosis. (A) Statistics showing pathological injury in the pancreas of mice in different groups. (B) Serum levels of

inflammatory factors (IL-6, IL-1β, TNF-α, MCP-1) in different groups of mice. (C) Representative histological HE staining images in lung of mice in different groups. Scale bar=100 μm. (D) Representative histological HE staining images in kidney of mice in different groups. Scale bar =50 μm. * $P<0.05$, ** $P<0.01$, *** $P<0.001$. n=6 per group.

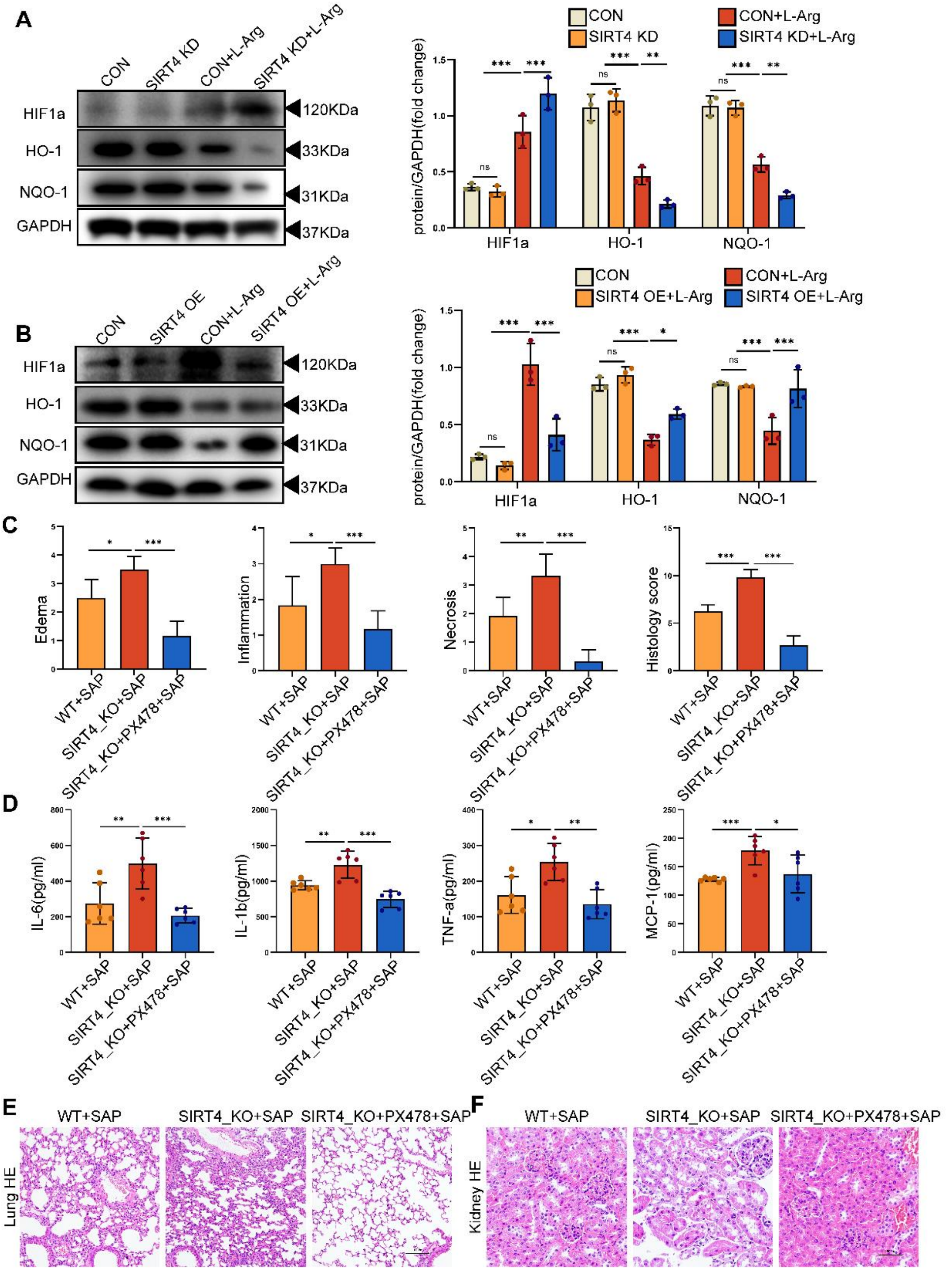

Fig. S4 The regulatory effect of SIRT4 on ferroptosis in SAP depends on the HIF-1α/HO-1 pathway. (A) The protein expression level of HIF-1α, HO-1 and NQO-1 in AR42J cells of SIRT4 KD experimental group. (B) The protein expression level of HIF-1α, HO-1 and NQO-1 in AR42J cells of SIRT4 OE experimental group. (C) Statistics showing pathological injury in the pancreas of mice in different groups. (D) Serum levels of inflammatory factors (IL-6, IL-1β, TNF-α and MCP-1) in different groups of mice after using HIF-1α inhibitor PX478 (100 mg/kg). (E) Representative histological HE staining images in lung of mice in different groups after using HIF-1α inhibitor PX478 (100 mg/kg). Scale bar =100 μm. (F) Representative histological HE staining images in kidney of mice in different groups after using HIF-1α inhibitor PX478 (100 mg/kg). Scale bar =50 μm. A-F used GAPDH as the reference protein. * $P<0.05$, ** $P<0.01$, *** $P<0.001$, ns., not significant. n=6 per group.

Supplemental Table 1 The links for the kits used in this study.

Reagent name	Purchase channels	The links
FOREGENE mouse tail gene identification box	Chengdu Fuji Biotechnology Co., Ltd. (Chengdu, China)	http://www.foregene.com/scientific_detail.aspx?t=19&pid=4&cid=78
Fer-1 (HY-100579)	MedChemExpress Corporation (Shanghai, China)	https://www.medchemexpress.cn/Ferrostatin-1.html
PX478（HY-10231）	MedChemExpress Corporation (Shanghai, China)	https://www.medchemexpress.cn/PX-478.html
Amylase ELISA kit (mlo27545)	Shanghai Enzyline Biotechnology Co., Ltd. (Shanghai, China)	https://www.mlbio.cn/goods-27545.html
Lipase ELISA kit (A054-2-1)	Nanjing Construction Institute of Biological Engineering (Nanjing, China)	http://www.njjcbio.com/products.asp?id=2740
MCP-1 ELISA Kit (EMC113.96)	Xinsheng Biotech Co., Ltd. (Shenzhen, China)	http://www.neobioscience.com/prod_view.aspx?TypeId=591&Id=1364139&FId=t3:591:3
IL-6 ELISA Kit (EMC004.96)	Xinsheng Biotech Co., Ltd. (Shenzhen, China)	http://www.neobioscience.com/prod_view.aspx?TypeId=591&Id=1364552&FId=t3:591:3
IL-1β ELISA Kit (EMC001b.96)	Xinsheng Biotech Co., Ltd. (Shenzhen, China)	http://www.neobioscience.com/prod_view.aspx?TypeId=591

		&Id=1364429&FId=t3:591:3
TNF-α ELISA Kit (EMC102a.96)	Xinsheng Biotech Co., Ltd. (Shenzhen, China)	http://www.neobioscience.com/prod_view.aspx?TypeId=591&Id=1363838&FId=t3:591:3
MDA kit (A003-1-2)	Nanjing Construction Institute of Biological Engineering (Nanjing, China)	http://www.njjcbio.com/products.asp?id=287
Total SOD kit (A001-1-1)	Nanjing Construction Institute of Biological Engineering (Nanjing, China)	http://www.njjcbio.com/products.asp?id=283
Trace and prototype GSH kit (A006-2-1)	Nanjing Construction Institute of Biological Engineering (Nanjing, China)	http://www.njjcbio.com/products.asp?id=1532
ROS determination kit (S0033)	Beyotime Biotechnology Co., Ltd. (Shanghai, China)	https://www.beyotime.com/product/S0033S.htm
Hydrogen peroxide (H_2O_2) detection kit (S0038)	Beyotime Biotechnology Co., Ltd. (Shanghai, China)	https://www.beyotime.com/product/S0038.htm

Supplemental Table 2 Primer sequences used in RT-PCR experiments.

**Primer Name**	**Primer sequences**
SIRT4	Forward: ATGTGGATGCTTTGCACACCAAGG<br>Reverse: TTCAGGACTTGGAAACGCTCTTGC
GAPDH	Forward: ATGTGGATGCTTTGCACACCAAGG<br>Reverse: TTCAGGACTTGGAAACGCTCTTGC
